# Global threat status, rarity, and species distribution affect prevalence of Atlantic Forest endemic birds in citizen-collected datasets

**DOI:** 10.1017/ext.2024.22

**Published:** 2024-11-22

**Authors:** Lucas Rodriguez Forti, Ana Marta P. R. da Silva Passetti, Talita Oliveira, Juan Lima, Arthur Queiros, Maria Alice Dantas Ferreira Lopes, Judit K. Szabo

**Affiliations:** 1 Departamento de Biociências, Universidade Federal Rural do Semi-Árido, Av. Francisco Mota, 572 – Bairro Costa e Silva, 59625-900, Mossoró Rio Grande do Norte, Brazil; 2 Programa de Pós-Graduação em Ecologia: Teoria, Aplicações e Valores, Instituto de Biologia, Universidade Federal da Bahia, Rua Barão de Jeremoabo, 668 – Campus de Ondina CEP: 40170-115 Salvador Bahia, Brazil; 3 Undergraduate program in Ecology, Universidade Federal Rural do Semi-Árido, Av. Francisco Mota, 572 – Bairro Costa e Silva, 59625-900, Mossoró Rio Grande do Norte, Brazil; 4 Programa de Pós-Graduação em Ecologia e Conservação, Universidade Federal Rural do Semi-Árido, Av. Francisco Mota, 572 – Bairro Costa e Silva, 59625-900, Mossoró Rio Grande do Norte, Brazil; 5 Undergraduate program in Veterinary Medicine, Universidade Federal Rural do Semi-Árido, Av. Francisco Mota, 572 – Bairro Costa e Silva, 59625-900, Mossoró Rio Grande do Norte, Brazil; 6 Research Institute for the Environment and Livelihoods, Charles Darwin University, Casuarina, Northern Territory 0909, Australia

**Keywords:** biodiversity hotspots, Brazil, citizen science, community science, monitoring

## Abstract

The Atlantic Forest is one of the most threatened biomes globally. Data from monitoring programs are necessary to evaluate the conservation status of species, prioritise conservation actions and to evaluate the effectiveness of these actions. Birds are particularly well represented in citizen-collected datasets that are used worldwide in ecological and conservation studies. Here, we analyse presence-only data from three online citizen science datasets of Atlantic Forest endemic bird species to evaluate whether the representation of these species was correlated with their global threat status, range and estimated abundance. We conclude that even though species are over- and under-represented with regard to their presumed abundance, data collected by citizen scientists can be used to infer species distribution and, to a lesser degree, species abundance. This pattern holds true for species across global threat status.

## Impact statement

Given the high rates of biodiversity loss globally, knowledge gaps need to be filled urgently in order to inform and prioritise conservation actions. Research and conservation are particularly important in tropical and megadiverse biomes, such as the Atlantic Forest. Given the lack of resources available for scientific research, professional scientists are struggling to conduct studies in these fragile biomes, particularly on long-term and at large scales. However, in the past two decades, nonprofessional scientists have been participating in the research process. Furthermore, data collected by these actors have been used in large-scale studies. Therefore, citizen science is becoming an important player in biodiversity knowledge production. Nevertheless, spatial, temporal and other biases resulting from unstructured sampling need to be understood and accounted for in order to make the collected data useful for decision-making. In this study, we evaluate how the estimated abundance, global threat status and spatial distribution of species affect the number of observations citizen scientists collect. We use endemic Brazilian Atlantic Forest bird species occurrence data from three online citizen science platforms. We found that threatened species were less frequently observed by citizen scientists than non-threatened species. Species with larger distribution ranges had more observations than species with more restricted ranges in all global threat status categories. In conclusion, citizen science data can be used to predict species distribution ranges, reducing knowledge gaps for Brazilian Atlantic Forest birds. Therefore, considering data contributed by citizen science can shorten the path to conservation actions.

## Introduction

Studies of the biodiversity of the Brazilian Atlantic Forest biome have resulted in important datasets of morphological traits and species abundance (Hasui et al., 2018; Rodrigues et al., 2019). Nevertheless, there is still a large Wallacean deficit with regard to the biodiversity of the biome (Colli‐Silva et al., 2020; Marques and Grelle, 2021). Species endemism is exceptionally high in the Atlantic Forest (Costa et al., 2000; da Silva et al., 2004; Cruz and Feio, 2007) and given the long history of deforestation (Dean, 2002) and the effects of climate change (Vale et al., 2018), this biome has been classified as one of the most threatened biodiversity hotspots on the planet and its exuberant flora and fauna are a constant source of concern for conservation biologists. In spite of ongoing restoration efforts (Romanelli et al., 2022), many endemic species still face a high risk of extinction (de Lima et al., 2020). Large data gaps plague up-to-date estimations of population size, dynamics and distribution of most species, making threat status assessments and conservation action prioritisation inaccurate. Threatened species tend to be rare and have a more restricted distribution than species evaluated as Least Concern on the global Red List by the International Union for Conservation of Nature (https://www.iucnredlist.org/). Species facing a higher risk of extinction often require broader actions and more intensive monitoring than less threatened taxa (Green and Young, 1993; Martikainen and Kouki, 2003).

Birds in particular are highly threatened in the Atlantic Forest – five to seven bird species have likely been driven to extinction in the wild in this biome and a further nine are Critically Endangered (Develey and Phalan, 2021). Fortunately, this group is also popular among observers, as besides paid scientists, 30–40,000 Brazilian birdwatchers are known to generate information for bird conservation (Develey, 2021).

Unlike “traditional” science, which is conducted by highly trained and paid personnel, community (or citizen) science data are contributed by volunteer members of the public (Louv and Fitzpatrick, 2012). These initiatives have become instrumental in generating monitoring data globally and at multiple scales (Chandler et al., 2017). In addition, observations often originate from private properties and other areas, which are not always accessible to professional researchers (Callaghan et al., 2021). Crowdsourcing through digital citizen science platforms has increased the rate of global biodiversity information production (Kelling et al., 2019). The number of occurrence records collected by volunteers within global databases, such as the Global Biodiversity Information Facility (GBIF; https://www.gbif.org/), has massively increased over time (Boakes et al., 2010; Petersen et al., 2021). Birds, in particular, have received the most interest from citizen scientists all around the world, and consequently, this group has the highest representation within GBIF (Troudet et al., 2017).

Currently, over 60% of all GBIF species occurrence data from Brazil are birds recorded through eBird (ebird.org). eBird accepts lists, photos or sound recordings of birds that observers see or hear while walking transects or through incidental observations. Observers also record geographic coordinates and the time and the day of the observation. The eBird database is curated by taxon specialists and the platform provides scientists and the interested public with real-time data on bird distributions and abundance. Other platforms have also become popular in Brazil and produce data on the distribution of birds in the country. For instance, WikiAves is a Brazilian website for birdwatchers, with the objective of supporting, disseminating and promoting birdwatching activities through photos and sound recordings, while helping with the identification of species and encouraging communication between observers. WikiAves accepts photos and sound recordings of bird species that occur in Brazil, but does not require exact coordinates of the observations, only the name of the municipality where the bird was recorded. Among other topics, this database has been used to study species distribution and migration (Cunha et al., 2022; Atwood, 2023), behaviour (Tubelis and Sazima, 2020; de Souza et al., 2022; Tubelis et al., 2022), diet (Schneider et al., 2023), and species interactions (Bosenbecker et al., 2023). A third platform with a high number of bird observations (270,888) in Brazil is iNaturalist. This generalist platform accepts photos and sound recordings of any organism and has been an important source of biodiversity data (Seregin et al., 2020; Mesaglio and Callaghan, 2021; Forti and Szabo, 2023). In this platform, artificial intelligence suggests identification for the submitted images and other users, including taxon experts, also contribute with their knowledge. Providing exact geographic coordinates makes it possible to use iNaturalist observations in a wide range of scientific studies, enabling spatial analyses and inferring relationships between organisms and their habitats, climate and other characteristics (Forti et al., 2022a, 2022b).

The increasing number of occurrence records collected by citizen scientists reflects a combination of increased public awareness and participation in citizen science initiatives and new technologies for recording and submitting observations (Chandler et al., 2017; Mihoub et al. 2017). At the same time, the mobilisation of other data sources, such as museum collections and the published literature has also increased the number of occurrence records in GBIF (Boakes et al. 2010). The combination of these data sources has allowed robust studies in the area of biogeography and macroecology (Liu et al., 2022; Moles and Xirocostas, 2022; Martinez et al., 2023).

In ecological studies, the number of observed individuals is often used as a proxy for species abundance. However, observations submitted by volunteers are often biased spatially – more frequent in areas of high human population density (Di Cecco et al., 2021; Forti and Szabo, 2023), temporally – observers prefer months and days when they are free and when climatic conditions are favourable (Bowler et al., 2022) and by taxonomy – depending on species characteristics, such as body colour, size, and shape (Callaghan et al., 2021; Marcenò et al., 2021). Also, the behaviour and habitat preference of some species make recording them more difficult, demanding higher observer skills or more experience, and this can result in the underrepresentation of some species of conservation concern in citizen science datasets and numbers of observations that do not directly reflect true abundance (Szabo et al., 2012). In spite of these issues, unstructured data from eBird, WikiAves and iNaturalist have been used to support conservation decision-making (Schubert et al., 2019; Spear et al., 2023). In this context, understanding factors that affect the number of observations submitted by citizen scientists is important during data analysis and interpretation.

In this work, we study the relationship between the distribution (extent of occurrence) and estimated abundance and biomass of species with the number of observations made by citizen scientists. We focus on endemic birds of the Brazilian Atlantic Forest using data from three major citizen science platforms. We also evaluate the relationship between species distribution and the number of observations in relation to the global threat category. While these relationships may seem intuitive, the behaviour of observers can vary between regions and the composition of different observer profiles can change the patterns of the data collected by them (Tulloch and Szabo, 2012). Nevertheless, our hypothesis is that the number of bird observations in the datasets is a function of the threat status of the species, which, in turn, is affected by species rarity and trends, reflected by the extent of geographic distribution and the abundance of the species (IUCN, 2022). We also describe under-, and overrepresented species and list potential actions to fill knowledge gaps, particularly species occurrence and population trends, of Brazilian Atlantic Forest bird species. In addition, we suggest future directions for the use of citizen science data in biodiversity conservation in this highly threatened biome.

## Results

We identified a total of 1,204,210 observations of endemic birds from the Atlantic Forest that have been submitted by citizen scientists to the three platforms. After removing duplicate observations and restricting the dataset to 2000–2022, 838,880 observations remained, representing approximately 70% of the raw data.

**Figure 1.**
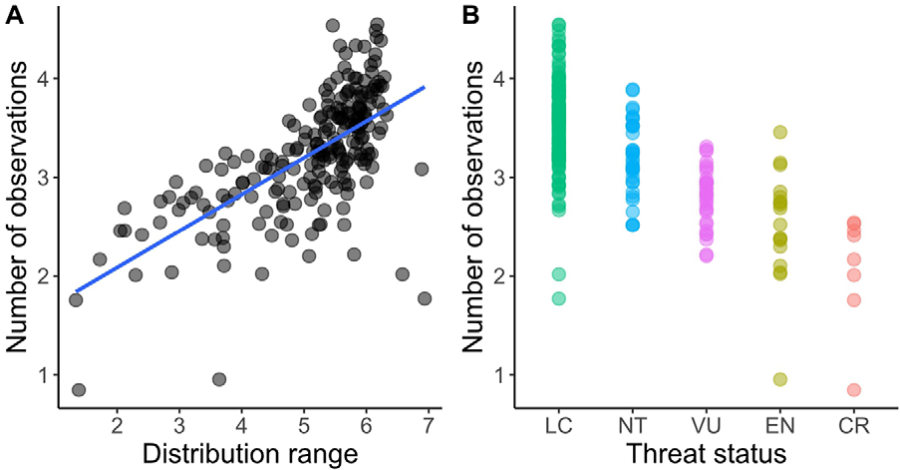
Number of observations of bird species endemic to the Atlantic Forest in Brazil in three citizen science platforms according to the distribution range of species (A) (both variables in log10 scale); and (B) the global threat status of the species (IUCN 2023).

**Figure 2.**
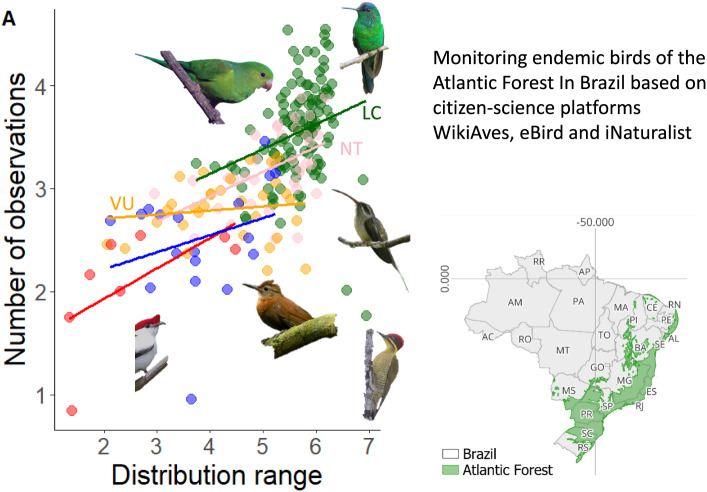
Number of observations of birds in the Atlantic Forest carried out by citizen scientists in relation to the distribution range of the species (both variables in log10 scale). Regression lines were calculated based on the global threat categories (IUCN): LC: Least Concern, NT: Near Threatened, VU: Vulnerable, EN: Endangered and CR: Critically Endangered. Species illustrated at the bottom of the graph are under-represented, such as the critically endangered *Merulaxis stresemanni* and *Antilophia bokermanni*, the Vulnerable *Sclerurus cearensis* and the Least Concern *Phaethornis malaris.* The species illustrated at the top of the graph, *Brotgeris tirica* and *Thalurania glaucopis* are overrepresented in the database. Images were provided by the following iNaturalist observers: @Anderson Sandro, @Luiz Alberto Santos, @Nereston Camargo, @Tomaz Melo, @Douglas Clarkee and @manequinho.

**Figure 3.**
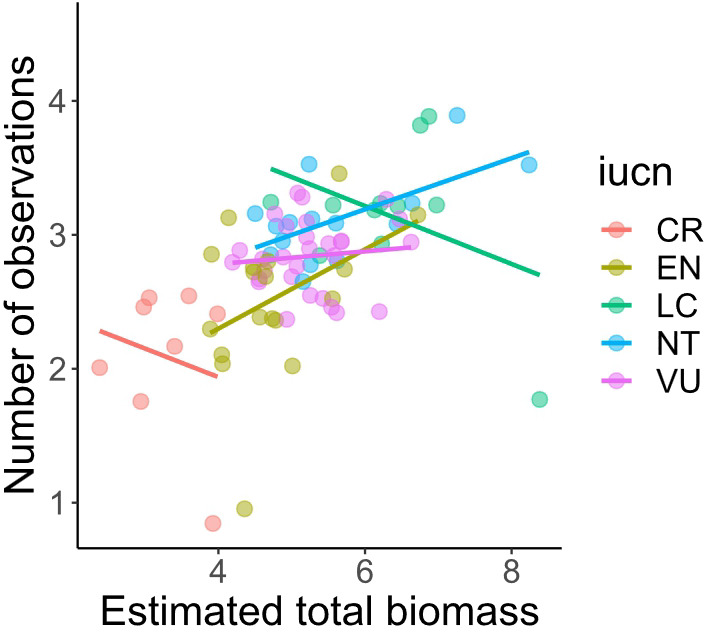
Number of observations of birds in the Atlantic Forest carried out by citizen scientists in relation to the estimated total biomass (both variables in log10 scale). Regression lines were calculated based on the global threat categories (IUCN): LC: Least Concern, NT: Near Threatened, VU: Vulnerable, EN: Endangered, and CR: Critically Endangered.

We found positive correlations between the range of species distribution, their extent of occurrence and their estimated abundance (see raw data in Supplementary Table 1). The size of the distribution range and threat status of the species affected the number of observations submitted by citizen scientists (Figure 1). The first mixed model (AIC = 3835.317; r^2^ = 0.50; REML = 255.6) had a positive value for the estimated coefficient (estimate = 0.21073; t-value = 5.312; p < 0.01) for the number of observations due to the range of species distributions, even considering the effects of family and threat status as random factors. These two random factors retained a large proportion of the variation in the residuals, and the value for threat status (SD_threat_ = 0.3027) was higher than the value for family (SD_family_ = 0.1473) with SD_residual_ = 0.4054. Nevertheless, the interaction between range and threat status was not significant in their effect on the number of observations (Supplementary Table 2). A larger distribution range seemed to result in more observations within threat categories (Figure 2). Based on the interaction term, IUCN status did not significantly influence the slope of the range size, hence the positive relationship held true across threat categories. In the second mixed model, estimated total biomass also had a significant effect on the number of observations made by citizen scientists (REML = 87.9, estimated coefficient = 0.209, and p = 0.004). However, based on a visual analysis of the residuals and the results of the third mixed effect model for estimated total biomass controlled only by IUCN status, it had a worse fit than the previous model (REML = 106.8, estimated coefficient = 0.1379, r^2^ = 0.329, and p = 0.018), with different patterns for different threat categories. The effect was negative for Critically Endangered and Least Concern species and positive for the rest, i.e., higher estimated biomass led to more observations (Figure 3).

**Figure 4.**
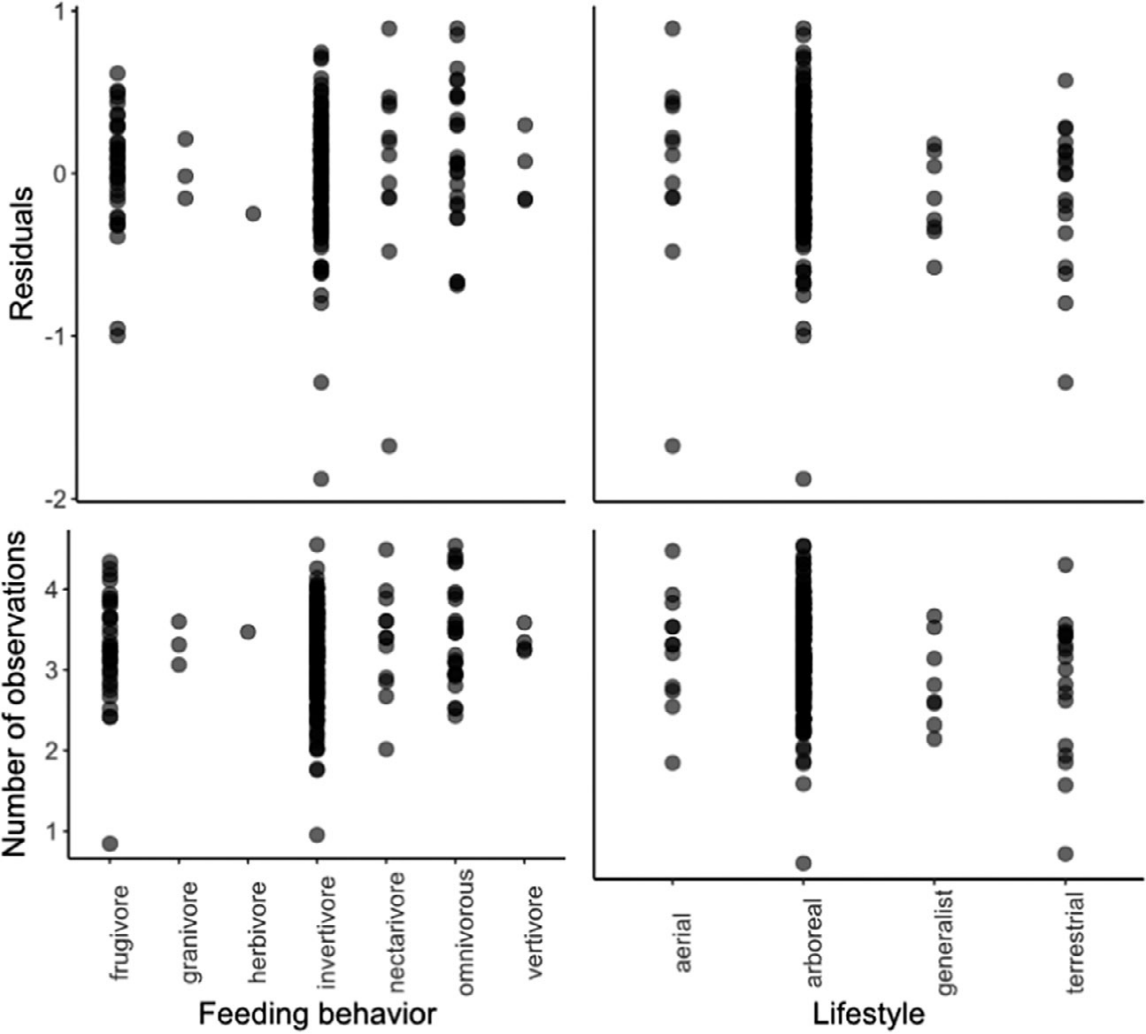
Number of observations made by citizen scientists of birds with different feeding behaviour (top left) and vertical strata (top right) and the distribution of model residuals for different categories of feeding behaviour (bottom left) and vertical strata (bottom right) for Atlantic Forest endemic birds. The number of observations and the residuals are shown at a logarithmic scale.

Some Least Concern species, such as the Golden-green Woodpecker (*Piculus chrysochloros*; r = – 1,878) and the Great-billed Hermit (*Phaethornis malaris*; r = – 1,674; Supplementary Table 1) deviated from the model prediction by having negative residuals and were under-represented in the citizen science data. Certain threatened species also had lower than predicted representation, including the Endangered Boa Nova Tapaculo (*Scytalopus gonzagai*; r = – 1,284); and the Critically Endangered Araripe Manakin (*Antilophia bokermanni*; r = – 0.998). On the other hand, common Least Concern species, such as the Plain Parakeet (*Brotogeris tirica*) and the Ruby-crowned Tanager (*Tachyphonus coronatus*) were overrepresented, both with r = 0.892. Some threatened species were also overrepresented in the dataset, such as the Critically Endangered Orange-bellied Antwren (*Terenura sicki*; r = 0.352), the Endangered Vinaceous-breasted Amazon (*Amazona vinacea*; r = 0.464) and the Vulnerable Fork-tailed Tody-tyrant (*Hemitriccus furcatus*; r = 0.475). Feeding habits and behaviour of the species did not explain model deviations and did not directly affect the number of observations per species in the datasets (Figure 4).

## Discussion

Our results suggest that threatened species are less frequently observed by citizen scientists in the Brazilian Atlantic Forest than nonthreatened species. Specifically, the more threatened a species is, the fewer observations the database contains. Based on our model, this pattern is due to range size and potentially, to a lesser extent, population size. This pattern suggests that citizen science can provide useful data for assessing the population status of birds in the Atlantic Forest, mainly with regard to the distribution range of the species. Therefore, citizen science data can reflect changes in the spatial distribution of bird species in the Atlantic Forest.

In fact, 67% of threatened and Near Threatened species of the total 216 Atlantic Forest bird species reported in the three citizen science datasets show declining population trends, while only 5% have unknown trends (BirdLife International, 2023). Given that citizen scientists can have a preference for rare or threatened species as shown in data from Australia (Tulloch and Szabo, 2012), it is not always possible to use the number of observations as an approximation of the size or distribution of bird populations. Furthermore, dull-coloured and shy species can be underreported by casual observers (Szabo et al., 2012).

Another caution we must take when interpreting citizen science data is related to oversampling in urban areas – either due to increasing spatial sampling bias over time or environmental change pooled with constant spatial sampling bias. This bias can lead to an overestimation of declines in species that are negatively affected by urban cover (Bowler et al., 2022). This effect may also explain the worse performance of the alternative model that incorporated total biomass based on species abundance estimates. Many factors could affect the number of observations, and these effects can be stronger than the abundance of the species.

Studies assessing observer behaviour have also shown a taxonomic bias in the representativeness of species in citizen science datasets (Tulloch and Szabo, 2012; Callaghan et al., 2021). In fact, body size has been an important predictor of detectability, with larger animals seen more often than smaller ones (Callaghan et al., 2021). However, some threatened species had a relatively high representation in our dataset regardless of their body sizes and these species are known to be charismatic and the object of organised initiatives (Martinez and Prestes, 2021). One example is the Vinaceous-breasted Amazon (Zulian et al., 2021), which has been reintroduced into the wild in some areas and has been a coveted target for observers. Local and focal citizen science projects have also been successful, particularly those involving iconic species, such as the Toco Toucan (*Ramphastos toco*; Schaaf et al., 2024).

In general, habitat loss in the Atlantic Forest makes continued sampling by citizen scientists even more important. Within 20 to 30 years, unstructured databases are estimated to gain more importance as their use in population trend calculations will increase (Szabo et al., 2010). Gathering information from different data sources can help to separate species dynamics from spatial biases in sampling (Dorazio, 2014; Fithian et al., 2015; Pacifici et al. 2017). This information can support the simultaneous modelling of presence-only data and standardised or presence-absence data in integrated distribution models (Dorazio, 2014; Fithian et al., 2015; Pacifici et al. 2017). With protection, habitat recovery and restoration, ongoing monitoring is more important than ever (Develey and Phalan, 2021). Up-to-date population sizes and ranges can inform us whether these actions are sufficient, or whether other measures, such as predator control, translocations, or *ex situ* management need to be applied (Develey and Phalan, 2021). Bird observation has improved the attitude of the Brazilian public towards biodiversity, promoting bird conservation and increasing knowledge about the birds of Brazil (Develey, 2021).

Barriers to participating in citizen science have decreased over the past decade due to new outreach projects and smartphone apps, leading to greater inclusion of people with less experience, and this has apparently been happening at the national scale in Brazil (Forti and Szabo, 2023). Recently joined participants may differ in their recording behaviour and be less likely to visit remote places to record species compared to observers who have been active for decades, but even so, data contributed by people of different profiles are important to detect trends and monitor changes in species distribution. High public engagement in citizen science is crucial and initiatives involved in adaptive sampling that addresses spatial and temporal gaps, as well as taxonomic bias need to be supported (Callaghan et al., 2023). Educational projects using a citizen science approach can be particularly important in collecting data in undersampled regions (Forti, 2023).

As databases grow, we continue to learn about biases and errors in citizen science data, including identification errors (Gorleri and Areta, 2022; Gorleri et al., 2023). Nevertheless, the resulting large databases have created enormous opportunities for ecologists to address questions about biodiversity patterns at large spatial scales (Theobald et al. 2015). Developments in statistical modelling also enable us to explain many of the biases and sources of heterogeneity in unstructured data (Isaac et al. 2014). The lack of standardised long-term monitoring for most taxa also increases the value of these datasets when assessing species turnover in ecological communities over time.

When planning citizen science initiatives, sample representativeness should be maximised (Callaghan et al., 2023). Local residents (as opposed to visitors) should be encouraged to survey the birds, as repeated surveys add value to monitoring also this type of volunteer is known to visit “less exciting” locations and record common species (Tulloch and Szabo, 2012). Citizen science data can also be better integrated with structured surveys conducted by professional scientists, in the sense that monitoring through standardised surveys should focus on the gaps left by volunteers (Tulloch et al., 2013).

## Conclusions

As the observation patterns identified for Atlantic Forest endemic birds might not be representative of all taxonomic groups in this biome, further studies should focus on the contribution of citizen science to observations of other taxa at a large scale. Our results suggest that citizen science initiatives contribute to our knowledge about endangered species in the biome in a meaningful way and this approach is expected to become even more relevant in the future for decision-making involving rare and/or threatened species.

## Methods

Study Area: Our study was conducted in the Atlantic Forest, which is the second largest tropical forest in South America behind the Amazon. The Atlantic Forest extends along the entire Brazilian coast and contains large human population centres, such as São Paulo, Rio de Janeiro and Salvador (Marques and Grelle, 2021). A complex topography covers a wide range of elevations from sea level to almost 3000 m a.s.l. and different substrates contribute to an intricate vertical stratification, creating microhabitats for a highly diverse biota (Morellato et al., 2000; Ramalho, 2004). The vegetation of this biome is a complex of evergreen, deciduous and semi-deciduous forests, along with mangroves, dunes and high-altitude fields (Ribeiro et al., 2011). These characteristics resulted in centres of endemism for multiple taxa and made the Atlantic Forest highly biodiverse (da Silva and Casteleti, 2003; Cardoso da Silva et al., 2004). Given the large extension of the Atlantic Forest, many areas are still poorly studied (Marques and Grelle, 2021). Starting with the Portuguese colonisation of Brazil, almost 500 years ago, anthropogenic pressures reduced the extent of native vegetation in the Atlantic Forest to 7.6% of its original extent (Marques and Grelle, 2021). Deforestation rates were historically driven by clearing for sugar cane and coffee plantations (Dean, 2002). Although habitat destruction has slowed down, climate change and the fragmentation of forest remnants still represent a major threat to the biodiversity of this biome (SOS Mata Atlântica/INPE, 2018; de Lima et al., 2020). In spite of many recent reforestation initiatives (https://pactomataatlantica.org.br/), endemic bird species are still declining (Develey and Phalan, 2021). The extent of protected areas is also relatively low, only covering 2% of the original area with native vegetation (Tabarelli et al., 2005).

Proceedings: To evaluate the representativeness of citizen science data, we extracted metadata from the three most important citizen science platforms in Brazil for 216 endemic birds of the Atlantic Forest (Vale et al., 2018). For species taxonomy, we followed BirdLife International (2023). The list of species and their status are detailed in Supplementary Table 1. We obtained data through formal requests to the Application Programming Interface (API) of eBird (https://ebird.org/home) and iNaturalist (https://www.inaturalist.org/), and compiled metadata using the Instant Data Scraper application (https://webrobots.io/instantdata/) from WikiAves (https://www.wikiaves.com.br/index.php). From iNaturalist, we only considered “research grade” observations, i.e., records validated at the species level with a consensus from at least 2/3 of the identifiers. We manually obtained population trends from BirdLife International’s Data Zone (2023). In the case of birds, BirdLife International is also the official assessor of IUCN Red List status. We obtained the IUCN global threat classification of species through the *rredlist* package (Chamberlain, 2020) in R version 4.2.1 (R Core Development Team, 2022). We included species from the following categories: Extinct (EX) for species, where there is no reasonable doubt that the last individual has died; extinct in the wild (EW), for species considered extinct in their natural habitat; Critically Endangered (CR), Endangered (EN), and Vulnerable (VU), following quantitative criteria designed to reflect varying degrees of threat of extinction (taxa in any of these three categories are collectively referred to as “threatened” henceforth); Near Threatened (NT), which is applied to species that currently do not meet the criteria for threatened, but are close to it or are likely to become threatened if ongoing conservation actions are reduced, interrupted or ceased; and Least Concern, for species that do not qualify (and are not close to qualifying) as threatened or Near Threatened. The category Least Concern indicates that, in terms of extinction risk, these species are of lower concern than species in other threat categories and does not imply that these species are not of conservation concern. None of the birds of the Atlantic Forest were classified by IUCN as Data Deficient or Not Evaluated. Five quantitative criteria are used to determine the threat category of a particular species, based on biological indicators of threatened populations, such as rapid population decline or reduced population size. These five criteria are as follows:

- Population size reduction (past, present and/or projected);
- Size of geographic distribution and fragmentation, few locations conditioned to threat, decline or fluctuations;
- Small population size with decline and fragmentation, fluctuations or few subpopulations;
- Population size too small or distribution too narrow;
- Quantitative Extinction Risk Analysis (e.g., Population Feasibility Analysis)

We obtained minimum and maximum abundance estimates and the extent of occurrence (EOO) of each species from BirdLife International (https://www.birdlife.org/datazone). As another metric, we obtained the size of the distribution range, which indicates the total area of the mapped range for the species from AVONET (Tobias et al., 2022). These numbers were calculated based on BirdLife International maps, considering areas, where a particular species was coded as present and distinguishing native and reintroduced ranges and areas, where the species was resident or visitor. We also collated data on body mass, feeding behaviour and life history traits (arboreal, aerial, etc.) for each of the species based on Tobias et al. (2022).

The area of occurrence (AOO) represents the geographical range of a species, which is calculated using a minimum convex polygon based on observation locations (Gaston, 1991). This metric is essential to evaluate a taxon based on Criterion B and can be used in Criterion A, which is used to assess changes in the distribution of a species (IUCN, 2022).

Data analysis: Statistical analyses and graphical visualisations were produced using R version 4.2.1 (R Core Development Team, 2022). We checked the heterogeneity of the dataset (abundance pattern based on the number of observations by each species) by applying Benford’s Law (Szabo et al., 2023) and found it to have marginal conformity with regard to the distribution of digits, which means that the data are of satisfactory quality (Forti et al., 2024). We produced graphs using the *ggplot2* package (Wickham, 2016).

We excluded one species, the Alagoas Curassow (*Mitu mitu*), from all analyses. Until its recent reintroduction in 2019 (Francisco et al., 2021), this species had the previous (unconfirmed) sighting in the wild in the late 1980s and is still considered EW by BirdLife International (2023). While our citizen-collected dataset contained two observations, there are no population size or range estimates provided by BirdLife International (2023).

As IUCN status is calculated over three generations or 20 years, we limited our database to observations made between January 1, 2000 and December 31, 2022. We also eliminated duplicate observations of the same species that occurred at the same geographic location on the same day using the function distinct from the *dplyr* package (Wickham et al., 2022). To obtain a more realistic estimate of species abundance, we calculated the median from the minimum and maximum values obtained from the Data Zone interface of BirdLife International (2023). We calculated total biomass by multiplying the median estimate of species abundance by the body mass of the species (Tobias et al., 2022).

We used three generalised mixed models through the *lme4* (Bates et al., 2015) and *lmerTest* (Kuznetsova et al., 2017) packages to assess the effect of species abundance, biomass and EOO, and distribution on the number of observations submitted by citizen scientists. First, we checked the correlation between fixed factors before fitting the models to avoid collinearity and log10 transformed all numerical variables to eliminate discrepancies in the dataset. Since the data were not normally distributed, we used the *cor.test* function through Spearman’s method to correlate the logarithm of the median abundance with the logarithm of the EOO and the distribution of the species. As the logarithm of the distribution range was highly correlated with species abundance and the EOO (Supplementary Table 3), we included it as a fixed factor in the first model, assigning the logarithm of the number of observations as the dependent variable and family and IUCN status as random factors. We used these two variables as random factors because both were correlated with the distribution range and estimated total biomass (Supplementary Table 4). In the second mixed model, we fit base 10 logarithm of the total estimated biomass of the species as a fixed factor and the same condition as in the first model for the random factors and the dependent variable. Then, we fit a third mixed model to isolate the effects of biomass on the number of observations controlled by IUCN status, using IUCN status as a random factor. No singular fit problems were identified for these models. We assessed the normality of the residuals visually using the *qqnorm* and *qqline* functions. After fitting the models, we obtained the residual maximum likelihood value (REML) and annotated the estimates of each fixed effect, as well as their significance value. We calculated variation around the estimates using a 95% confidence interval through the *confit.merMod* function. We checked indices of model performance and singularity using the *model_performance* and *check_singularity* functions of the *performance* package (Lüdecke et al., 2021). We saved residuals from the first mixed model to identify species that were under and overrepresented in the database. These residuals and the logarithm of the number of observations were also used to test possible effects of feeding behaviour and life history by graph visualisation. Finally, we fit a generalised linear model using the *lm* function to test the effect of the logarithm of the distribution range of species interacting with IUCN threat status. This procedure also allowed us to understand the effect of the logarithm of the distribution range of species on the logarithm of the number of observations within each threat status group. For the R script with the codes for all statistical models described above see Supplementary Material 5.

## Supporting information

Forti et al. supplementary material Forti et al. supplementary material

## Data Availability

The raw data for this study are available at https://zenodo.org/record/7775610#.ZCHnGnbMLb0.

## Supplementary material

The supplementary material for this article can be found at http://doi.org/10.1017/ext.2024.22.

## References

[r1] Atwood, JL (2023) Seasonal patterns of least tern distribution along the Atlantic Coasts of North, Central, and South America. Waterbirds 46 (1), 85–90. 10.1675/063.046.0111.

[r2] Bates DM, Maechler M, Bolker BM and Walker S (2015) Fitting linear mixed‐effects models using lme4. Journal of Statistical Software 67 (1), 1–48.

[r3] BirdLife International (2023) Birdlife Data Zone. http://datazone.birdlife.org/. Available at http://www.birdlife.org/datazone/speciessearchresults.php?reg=0&cty=13&cri=&fam=0&gen=0&spc=&cmn=&hab=&thr=&bt=&rec=N&vag=N&hdnAction=ADV_SEARCH&SearchTerms= (accessed 10 March 2023).

[r4] Bosenbecker, C, Amaral Anselmo P, Andreoli RZ, Shimizu GH, Oliveira PE, and Maruyama PK (2023) Contrasting nation-wide citizen science and expert collected data on hummingbird–plant interactions. Perspectives in Ecology and Conservation 21, 164–171. 10.1016/j.pecon.2023.03.004.

[r5] Bowler DE, Callaghan CT, Bhandari N, Henle K, Barth MB, Koppitz C, Klenke R, Winter M, Jansen F, Bruelheide H and Bonn A (2022) Temporal trends in the spatial bias of species occurrence records. Ecography 8, e06219. 10.1111/ecog.06219.

[r75] Boakes, EHl, McGowan, PJK, Fuller RA, Chang-qing D, Clark NE, O’Connor K, Mace GM (2010) Distorted views of biodiversity: Spatial and temporal bias in species occurrence PLoS Biology 8(6), e1000385 20532234 10.1371/journal.pbio.1000385 PMC2879389

[r6] Callaghan CT, Poore AGB, Hofmann M, Roberts CJ and Pereira HM (2021) Large‑bodied birds are over‑represented in unstructured citizen science data. Scientific Reports 11, 19073. 34561517 10.1038/s41598-021-98584-7 PMC8463711

[r7] Callaghan CT, Poore AGB, Mesaglio T, Moles AT, Nakagawa S, Roberts C, Rowley JJL, Vergés A, Wilshire JH and Cornwell WK (2021) Three frontiers for the future of biodiversity research using citizen science data. BioScience 71(1), 55–63.

[r8] Callaghan CT, Thompson MM, Woods A, Poore AGB, Bowler DE, Samonte F, Rowley JJL, Roslan N, Kingsford RT, Cornwell WK and Major RE (2023) Experimental evidence that behavioral nudges in citizen science projects can improve biodiversity data. BioScience 73, 302–313. 10.1093/biosci/biad012.

[r9] Cardoso da Silva JM, Cardoso de Sousa M and Castelletti CHM (2004) Areas of endemism for passerine birds in the Atlantic Forest, South America. Global Ecology and Biogeography 13, 85–92.

[r10] Chamberlain S (2020) rredlist: ‘IUCN’ Red List Client. R package version 0.7.0, https://CRAN.R-project.org/package=rredlist.

[r11] Chandler M, See L, Copas K, Bonde AM, López BC, Danielsen F, Legind JK, Masinde S, Miller-Rushing AJ, Newman G and Rosemartin A (2017) Contribution of citizen science towards international biodiversity monitoring. Biological Conservation, 213, 280–294.

[r12] Colli‐Silva M, Reginato M, Cabral A, Forzza RC and Vasconcelos TNdC (2020) Evaluating shortfalls and spatial accuracy of biodiversity documentation in the Atlantic Forest, the most diverse and threatened Brazilian phytogeographic domain. Taxon 69(3), 567–577. 10.1002/tax.12239

[r13] Costa LP, Leite YLR, da Fonseca GAB and da Fonseca MT (2000) Biogeography of South American Forest Mammals: endemism and diversity in the Atlantic Forest. Biotropica 32(4b), 872–881. 10.1111/j.1744-7429.2000.tb00625.x

[r14] Cruz CAG and Feio RA (2007) Endemismos em Anfíbios em Áreas de Altitude na Mata Atlântica no Sudeste do Brasil. In Nascimento LB and Oliveira ME (eds), Herpetologia no Brasil II: 117–126. Sociedade Brasileira de Herpetologia, Belo Horizonte.

[r15] Cunha, FCR, Esteves Lopes L, and Selezneva A (2022) Revealing migration schedule and potential breeding grounds of Lined Seedeaters using citizen science data. Emu 122 (3–4), 167–175. 10.1080/01584197.2022.2105235

[r16] da Silva, JMC and Casteleti CHM (2003) Status of the biodiversity of the Atlantic Forest of Brazil. In The Atlantic Forest of South America: biodiversity status, trends, and outlook., Carlos Galindo-Leal and Ibsen de Gusmao Câmara. 43–59. Washington, D.C.: Center for Applied Biodiversity Science and Island Press.

[r17] da Silva, JMC, de Sousa MC and Casteleti CHM (2004) Areas of endemism for passerine birds in the Atlantic Forest, South America. Global Ecology and Biogeography 13, 85–92.

[r18] de Lima RAF, Oliveira AA, Pitta GR, de Gasper AL, Vibrans AC, Chave J, Ter Steege H and Prado PI (2020) The erosion of biodiversity and biomass in the Atlantic Forest biodiversity hotspot. Nature Commucations 11(1), 1–16. 10.1038/s41467-020-20217-w PMC7733445 33311511

[r19] de Souza E, Lima‑Santos J, Entiauspe‑Neto OM, dos Santos MM, de Moura PR and Hingst‑Zaher E (2022) Ophiophagy in Brazilian birds: a contribution from a collaborative platform of citizen science. Ornithology Research 30, 15–24.

[r20] Dean W (2002) A Ferro e Fogo: A História da Devastação da Mata Atlântica Brasileira. São Paulo: Cia das Letras.

[r21] Develey, PF and Phalan BT (2021) Bird extinctions in Brazil’s Atlantic Forest and how they can be prevented. Frontiers in Ecology and Evolution 9, 624587.

[r22] Develey, PF (2021) Bird conservation in Brazil: challenges and practical solutions for a key megadiverse country. Perspectives in Ecology and Conservation 19, 171–178.

[r23] Di Cecco GJ, Barve V, Belitz MW, Stucky BJ, Guralnick RP and Hurlbert AH (2021) Observing the observers: how participants contribute data to iNaturalist and implications for biodiversity science. BioScience 71(11), 1179–1188.

[r24] Dorazio RM (2014). Accounting for imperfect detection and survey bias in statistical analysis of presence‐only data. Global Ecology and Biogeography, 23(12), 1472–1484.

[r25] Fithian W, Elith J, Hastie T and Keith DA (2015) Bias correction in species distribution models: pooling survey and collection data for multiple species. Methods in Ecology and Evolution, 6(4), 424–438. 27840673 10.1111/2041-210X.12242 PMC5102514

[r26] Forti LR (2023) Students as citizen scientists: project-based learning through the iNaturalist platform could provide useful biodiversity data. Biodiversity 24(1–2), 76–78. 10.1080/14888386.2023.2174595

[r27] Forti LR, Hepp F, de Souza JM, Protazio A and Szabo JK (2022a) Climate drives anuran breeding phenology in a continental perspective as revealed by citizen‐collected data. Diversity and Distributions, 2094–2109. 10.1111/ddi.13610

[r28] Forti LR, Retuci Pontes M, Augusto-Alves G, Martins A, Hepp F and Szabo JK (2022b) Data collected by citizen scientists reveal the role of climate and phylogeny on the frequency of shelter types used by frogs across the Americas. Zoology 155, 126052. 10.1016/j.zool.2022.126052. 36152596

[r29] Forti LR, Passetti A, Oliveira T, Lima J, Queiros A, Lopes MADF and Szabo JK (2024) Declining representation of imperiled Atlantic Forest birds in citizen-collected data. Perspectives in Ecology and Conservation. 10.1016/j.pecon.2024.02.001

[r30] Forti LR and Szabo JK (2023) The iNaturalist platform as a source of data to study amphibians in Brazil. Anais da Academia Brasileira de Ciências 95(1), e20220828. 10.1590/0001-3765202320220828 37075357

[r31] Francisco MR, Costa MC, Azeredo RMA, Simpson JGP, da Costa Dias T, Fonseca A, Pinto FJM and Silveira LF (2021) Recovered after an extreme bottleneck and saved by ex situ management: Lessons from the Alagoas curassow (Pauxi mitu [Linnaeus, 1766]; Aves, Galliformes, Cracidae). Zoo Biology 40(1), 76–78. 10.1002/zoo.21577 33107113

[r32] Gaston KJ (1991) How large is a species’ geographic range? Oikos 61(3), 434–438.

[r33] Gorleri FC and Areta JI (2022) Misidentifications in citizen science bias the phenological estimates of two hard-to-identify *Elaenia* flycatchers. Ibis 164(1), 13–26.

[r34] Gorleri FC, Jordan EA, Roesler I, Monteleone D and Areta JI (2023) Using photographic records to quantify accuracy of bird identifications in citizen science data. Ibis 165(2), 458–471.

[r35] Green RH and Young RC (1993) Sampling to detect rare species. Ecological Applications 3(2), 351–356. 27759323 10.2307/1941837

[r36] Hasui E, Metzger JP, Pimentel RG, Silveira LF, Bovo AADA, Martensen AC, Uezu A, Regolin AL, Bispo de Oliveira AA, Gatto CAFR, Duca C, Andretti CB, Banks-Leite C, Luz D, Mariz D, Alexandrino ER, de Barros FM, Martello F, Pereira IMDS, da Silva JN, Ferraz KMPMDB, Naka LN, Dos Anjos L, Efe MA, Pizo MA, Pichorim M, Goncalves MSS, Chaves Cordeiro PH, Dias RA, Muylaert RDL, Rodrigues RC, da Costa TVV, Cavarzere V, Tonetti VR, Silva WR, Jenkins CN, Galetti M and Ribeiro MC (2018) Atlantic Birds: a data set of bird species from the Brazilian Atlantic Forest. Ecology 99(2), 497. 29266462 10.1002/ecy.2119

[r80] Isaac NJB, van Strien A, August TA, de Zeeuw MP, Roy DB (2014) Statistics for citizen science: extracting signals of change from noisy ecological data. Methods in Ecology and Evolution 5, 1052–1060.

[r37] IUCN (2022) The IUCN red list of threatened species. Version 2022-1. https://www.iucnredlist.org (accessed on 1 September 2022).

[r81] IUCN (2023) The IUCN Red List of Threatened Species. Version 2023-1. https://www.iucnredlist.org. Accessed on 12 December, 2023.

[r38] Kelling S, Johnston A, Bonn A, Fink D, Ruiz-Gutierrez V, Bonney RE, Fernandez M, Hochachka WM, Julliard R, Kraemer R and Guralnick R (2019) Using semistructured surveys to improve citizen science data for monitoring biodiversity. BioScience 69, 170–179. 30905970 10.1093/biosci/biz010 PMC6422830

[r39] Kuznetsova A, Brockhoff PB and Christensen RHB (2017) lmerTest package: tests in linear mixed effects models. Journal of Statistical Software 82(13), 1–26.

[r40] Liu W, Smith DAS, Raina G, Stanforth R, Ng’Iru I, Ireri P, Martins DJ, Gordon IJ and Martin SH (2022) Global biogeography of warning coloration in the butterfly *Danaus chrysippus*. Biology Letters 18, 20210639. 10.1098/rsbl.2021.0639. 35642381 PMC9156917

[r41] Louv R and Fitzpatrick JW (2012) Citizen science: public participation in environmental research. Cornell University Press.

[r42] Lüdecke D, Ben-Shachar MS, Patil I, Waggoner P, and Makowski D (2021) Performance: an R package for assessment, comparison and testing of statistical models. Journal of Open Source Software 6 (60), 3139. 10.21105/joss.03139.

[r43] Marcenò C, Padullés Cubino J, Chytrý M, Genduso E, Salemi D, La Rosa A, Gristina AS, Agrillo E, Bonari G, Giusso del Galdo G, Ilardi V, Landucci F and Guarino R (2021) Facebook groups as citizen science tools for plant species monitoring. Journal of Applied Ecology 58(10), 2018–2028. 10.1111/1365-2664.13896.

[r44] Marques, MCM and Grelle CEV (2021) The Atlantic Forest: history, biodiversity, threats and opportunities of the mega-diverse forest. Springer Nature.

[r45] Martikainen P and Kouki J (2003) Sampling the rarest: threatened beetles in boreal forest biodiversity inventories. Biodiversity and Conservation 12, 1815–1831.

[r46] Martinez J and Prestes NP (2021) Biologia da conservação: Programa nacional para a conservação do Papagaio-de-peito-roxo e outras iniciativas. Livraria e Editora Werlang LTDA, Passo Fundo, Brazil.

[r47] Martinez N, Kirwan GM and Schweizer M (2023) Resolving disputed subspecies distribution limits, and revealing intraspecifc intergradation, in the Common Redstart *Phoenicurus phoenicurus* using citizen science and museum data. Journal of Ornithology 164, 1–12. 10.1007/s10336-022-02007-x

[r48] Mesaglio T and Callaghan CT (2021) An overview of the history, current contributions and future outlook of iNaturalist in Australia. Wildlife Research 48, 289–303.

[r82] Mihoub, J.-B., Henle, K., Titeux, N., Brotons, L., Brummitt, N. A., & Schmeller, D. S. (2017). Setting temporal baselines for biodiversity: the limits of available monitoring data for capturing the full impact of anthropogenic pressures. Scientific Reports, 7(1). 10.1038/srep41591 PMC5278508 28134310

[r49] Moles AT and Xirocostas ZA (2022) Statistical power from the people. Nature Ecology & Evolution 6, 1802–1803. 10.1038/s41559-022-01902-z 36266457

[r50] Morellato LPC, and Haddad CFB (2000) Introduction: the Brazilian Atlantic Forest. Biotropica 32 (4b): 786–792.

[r76] Petersen TK, Speed JDM, Grøtan V, Austrheim G (2021) Species data for understanding biodiversity dynamics: The what, where and when of species occurrence data collection. Ecological Solutions and Evidence 2. e12048.

[r78] Pacifici M, Visconti P, Butchart SHM, Watson JEM, Cassola FM, Rondinini C (2017) Species’ traits influenced their response to recent climate change. Nature Climate Change 7, 205–209.

[r51] R Core Development Team (2022) R: a language and environment for statistical computing. Foundation for Statistical Computing, Vienna, Austria. http://www.R-project.org/.

[r52] Ramalho, M (2004) Stingless bees and mass flowering trees in the canopy of Atlantic Forest: a tight relationship. Acta Botanica Brasileira 18 (1), 37–47.

[r53] Ribeiro, MC, Martensen AC, Metzger JP, Tabarelli M, Scarano FR, and Fortin M-J (2011) The Brazilian Atlantic Forest: a shrinking biodiversity hotspot. In Biodiversity hotspots, Frank E. Zachos and J. C. Habel. 405–434. Berlin, Heidelberg: Springer.

[r54] Rodrigues RC, Hasui É, Camara Assis J, Castro Pena JC, Muylaert RDL, Tonetti VR, Martello F, Regolin AL, Vernaschi Vieira da Costa T, Pichorim M, Carrano E, Lopes LE, Ferreira de Vasconcelos M, Suertegaray Fontana C, Langeloh Roos A, Gonçalves F, Banks-Leite C, Cavarzere V, Amorim Efe M, Alves MAS, Uezu A, Metzger JP, de Tarso Zuquim de Antas P, Paschoaletto Micchi de Barros Ferraz KM, Corsini Calsavara L, Bispo AA, Araujo HFP, Duca C, Piratelli AJ, Naka LN, Antunes Dias R, Gatto CAFR, Villegas Vallejos MA, dos Reis Menezes G, Bugoni L, Rajão H, Zocche JJ, Willrich G, Silveira da Silva E, Tonelli Manica L, Guaraldo AdC, Althmann G, Pereira Serafini P, Francisco MR, Lugarini C, Machado CG, Marques-Santos F, Bobato R, Arantes de Souza E, Donatelli RJ, Ferreira CD, Morante-Filho JC, Dantas Paes-Macarrão N, Macarrão A, Robalinho Lima M, Jacoboski LI, Candia-Gallardo C, Bejarano Alegre V, Jahn AE, de Camargo Barbosa KV, Cestari C, Nilton da Silva J, Stefanini Da Silveira N, Vara Crestani NAC, Peterle Petronetto A, Bovo AAA, Durão Viana A, Araujo AC, Hartuiq dos Santos A, Araújo do Amaral AC, Ferreira A, Vieira-Filho AH, Costa Ribeiro B, Missagia CCC, Bosenbecker C, Bronzato Medolago CA, Rodriguez Espínola CR, Faxina C, Campodonio Nunes CE, Prates C, Tomasio Apolinario da Luz D, Moreno DJ, Mariz D, Faria D, Meyer D, Doná EA, Alexandrino ER, Fischer EA, Girardi F, Borba Giese F, Santos Shibuya FL, Azevedo Faria F, Bittencourt de Farias F, de Lima Favaro F, Ferneda Freitas FJ, Chaves FG, Guedes Las-Casas FM, Rosa GLM, Massaccesi De La Torre G, Menezes Bochio G, Bonetti GE, Kohler G, Santos Toledo-Lima G, Piletti Plucenio G, Menezes Í, Denóbile Torres IM, Carvalho Provinciato IC, Réus Viana I, Roper JJ, Persegona JE, Barcik JJ, Martins-Silva J, Gava Just JP, Tavares-Damasceno JP, de Almeida Ferreira JR, Rodrigues Rosoni JR, Teixeira Falcon JE, Schaedler LM, Brioschi Mathias L, Deconto LR, da Cruz Rodrigues L, Afonso P. Meyer M, Repenning M, Melo MA, Santos de Carvalho MA, Rodrigues M, Conti Nunes MF, Ogrzewalska MH, Lopes Gonçalves M, Vecchi MB, Bettio M, Noronha da Matta Baptista M, Arantes MS, Ruiz NL, Bisetto de Andrade PG, Lima Ribeiro PH, Galetti Junior PM, Macario P, de Oliveira Fratoni R, Meurer R, Saint-Clair RS, Spilere Romagna R, Alves Lacerda RC, Serpa Cerboncini RA, Brioschi Lyra R, Lau R, Costa Rodrigues R, Rodrigues Faria R, Laps RR, Althoff SL, de Jesus S, Namba S, Vieira Braga T, Molin T, França Câmara TP, Rodrigues Enedino T, Wischhoff U, de Oliveira VC, Leandro-Silva V, Araújo-Lima V, de Oliveira Lunardi V, Farias de Gusmão R, de Souza Correia JM, Gaspar LP, Batista Fonseca RC, Fonseca Pires Neto PA, Medeiros Morato de Aquino AC, Betagni de Camargo B, Azevedo Cezila B, Marques Costa L, Montanheiro Paolino R, Zukeran Kanda C, Monteiro ECS, Oshima JEF, Alves-Eigenheer M, Pizo MA, Silveira LF, Galetti M and Ribeiro MC (2019) ATLANTIC BIRD TRAITS: a data set of bird morphological traits from the Atlantic Forests of South America. Ecology 100(6), e02647. 10.1002/ecy.2647 30845354

[r55] Romanelli JP, Meli P, Bispo Santos JP, Nogueira Jacob I, Rodrigues Souza L, Vieira Rodrigues A, Peruchi Trevisan D, Huang C, Almeida DRA, Silva LGM, Lopes Assad MLRC, Cadotte MW and Ribeiro Rodrigues R (2022) Biodiversity responses to restoration across the Brazilian Atlantic Forest. Science of the Total Environment 821, 153403. 10.1016/j.scitotenv.2022.153403 35101503

[r56] Schaaf AA, Haag LM, Gonzalez Baffa-Trasci NV, Yapura A, Chocobar N, Caldano SA, and Ruggera RA (2024) Comparing different citizen science platforms for collecting urban ecological data from Toco toucan (*Ramphastos toco*) in Argentina. Austral Ecology. 10.1111/aec.13459

[r57] Schneider LM, de Oliveira Santos C, Moreira-Lima L, and Hingst-Zaher E (2023) Peregrine falcon *Falco peregrinus* in Brazil: natural history through the lens of citizen science. Ornitologia Neotropical 34, 29–39. 10.58843/ornneo.v34i1.1121

[r58] Schubert SC, Tonelli Manica L and Guaraldo ADC (2019) Revealing the potential of a huge citizen-science platform to study bird migration. Emu 119(4), 364–373.

[r59] Seregin AP, Bochkov DA, Shner JV, Garin EV, Pospelov IN, Prokhorov VE, Golyakov PV, Mayorov SR, Svirin SA, Khimin AN, Gorbunova MS, Kashirina ES, Kuryakova OP, Bolshakov BV, Ebel AL, Khapugin AA, Mallaliev MM, Mirvoda SV, Lednev SA, Nesterkova DV, Zelenova NP, Nesterova SA, Zelenkova VN, Vinogradov GM, Biryukova OV, Verkhozina AV, Zyrianov AP, Gerasimov SV, Murtazaliev RA, Basov YM, Marchenkova KY, Vladimirov DR, Safina DB, Dudov SV, Degtyarev NI, Tretyakova DV, Chimitov DG, Sklyar EA, Kandaurova AN, Bogdanovich SA, Dubynin AV, Chernyagina OA, Lebedev AV, Knyazev MS, Mitjushina IY, Filippova NV, Dudova KV, Kuzmin IV, Svetasheva TY, Zakharov VP, Travkin VP, Magazov YO, Teploukhov VY, Efremov AN, Deineko OV, Stepanov VV, Popov ES, Kuzmenckin DV, Strus TL, Zarubo TV, Romanov KV, Ebel AL, Tishin DV, Arkhipov VY, Korotkov VN, Kutueva SB, Gostev VV, Krivosheev MM, Gamova NS, Belova VA, Kosterin OE, Prokopenko SV, Sultanov RR, Kobuzeva IA, Dorofeev NV, Yakovlev AA, Danilevsky YV, Zolotukhina IB, Yumagulov DA, Glazunov VA, Bakutov VA, Danilin AV, Pavlov IV, Pushay ES, Tikhonova EV, Samodurov KV, Epikhin DV, Silaeva TB, Pyak AI, Fedorova YA, Samarin ES, Shilov DS, Borodulina VP, Kropocheva EV, Kosenkov GL, Bury UV, Mitroshenkova AE, Karpenko TA, Osmanov RM, Kozlova MV, Gavrilova TM, Senator SA, Khomutovskiy MI, Borovichev EA, Filippov IV, Ponomarenko SV, Shumikhina EA, Lyskov DF, Belyakov EA, Kozhin MN, Poryadin LS and Leostrin AV (2020) “Flora of Russia” on iNaturalist: a dataset. Biodiversity Data Journal 8, e59249. 10.3897/BDJ.8.e59249 33244292 PMC7686226

[r60] SOS Mata Atlântica/INPE (2018) Atlas dos remanescentes florestais da Mata Atlântica: período 2016–2017 – relatório técnico. São Paulo: SOS Mata Atlântica/INPE.

[r61] Spear D, van Wilgen NJ, Rebelo AG and Botha JM (2023) Collating biodiversity occurrence data for conservation. Frontiers in Ecology and Evolution 11, 1037282. 10.3389/fevo.2023.1037282.

[r62] Szabo JK, Forti LR and Callaghan CT (2023) Large biodiversity datasets conform to Benford’s law: implications for assessing sampling heterogeneity. Biological Conservation 280, 109982. 10.1016/j.biocon.2023.109982

[r63] Szabo JK, Fuller RA and Possingham HP (2012) A comparison of estimates of relative abundance from a weakly structured mass-participation bird atlas survey and a robustly designed monitoring scheme. Ibis 154, 468–479.

[r64] Szabo JK, Vesk PA, Baxter PWJ and Possingham HP (2010) Regional avian species declines estimated from volunteer-collected long-term data using List Length Analysis. Ecological Applications 20(8), 2157–2169. 10.1890/09-0877.1 21265449

[r65] Tabarelli, ML, Pinto P, Silva JMC, Hirota MM, and Bedê LC (2005) Desafios e oportunidades para a conservação da biodiversidade na Mata Atlântica brasileira. Megadiversidade 1 (1), 132–138.

[r66] Tobias JA, Sheard C, Pigot AL, Devenish AJM, Yang JY, Sayol F, Neate-Clegg MHC, Alioravainen N, Weeks TL, Barber RA, Walkden PA, MacGregor HEA, Jones SEI, Vincent C, Phillips AG, Marples NM, Montaño-Centellas FA, Leandro-Silva V, Claramunt S, Darski B, Freeman BG, Bregman TP, Cooney CR, Hughes EC, Capp EJR, Varley ZK, Friedman NR, Korntheuer H, Corrales-Vargas A, Trisos CH, Weeks BC, Hanz DM, Töpfer T, Bravo GA, Remeš V, Nowak L, Carneiro LS, Moncada AJ, Matysioková B, Baldassarre DT, Martínez-Salinas A, Wolfe JD, Chapman PM, Daly BG, Sorensen MC, Neu A, Ford MA, Mayhew RJ, Silveira LF, Kelly DJ, Annorbah NND, Pollock HS, Grabowska-Zhang AM, McEntee JP, Gonzalez JCT, Meneses CG, Muñoz MC, Powell LL, Jamie GA, Matthews TJ, Johnson OW, Brito GRR, Zyskowski K, Crates R, Harvey MG, Jurado Zevallos M, Hosner PA, Bradfer‐Lawrence T, Maley JM, Gary Stiles F, Lima HS, Provost KL, Chibesa M, Mashao M, Howard JT, Mlamba E, Chua MAH, Li B, Gómez MI, García NC, Päckert M, Fuchs J, Ali JR, Derryberry EP, Carlson ML, Urriza RC, Brzeski KE, Prawiradilaga DM, Rayner MJ, Miller ET, Bowie RCK, Lafontaine R-M, Scofield RP, Lou YQ, Somarathna L, Lepage D, Illif M, Neuschulz EL, Templin M, Dehling DM, Cooper JC, Pauwels OSG, Analuddin K, Fjeldså J, Seddon N, Sweet PR, DeClerck FAJ, Naka LN, Brawn JD, Aleixo A, Böhning-Gaese K, Rahbek C, Fritz SA, Thomas GH and Schleuning M (2022) AVONET: morphological, ecological and geographical data for all birds. Ecology Letters 25, 581–597. 10.1111/ele.13898. 35199922

[r67] Tubelis DP, Araújo SK and Gomes TV (2022) First sighting of a snake as nuptial gift by a pair of Roadside Hawks, *Rupornis magnirostris* (Aves: Accipitridae), in the Brazilian dry forest region. Herpetology Notes 15, 817–819.

[r68] Tubelis DP and Sazima I (2020) Biologia reprodutiva do carão, *Aramus guarauna* (Gruiformes: Aramidae), no Pantanal brasileiro, com uso de dados da ciência-cidadã. Atualidades Ornitológicas 215, 8–11.

[r69] Tulloch A and Szabo JK (2012) A behavioural ecology approach to understand volunteer surveying for citizen science datasets. Emu 112, 313–325. 10.1071/MU12009

[r70] Tulloch AIT, Mustin K, Possingham HP, Szabo JK and Wilson KA (2013) To boldly go where no volunteer has gone before: predicting volunteer activity to prioritize surveys at the landscape scale. Diversity and Distributions 19, 465–480.

[r77] Troudet, J., Grandcolas, P., Blin, A., Vignes-Lebbe, R., & Legendre, F. (2017). Taxonomic bias in biodiversity data and societal preferences. Scientific Reports, 7(1). 10.1038/s41598-017-09084-6 PMC5567328 28831097

[r79] Theobald EJ, Ettinger AK, Burgess HK, DeBey LB, Schmidt NR, Froehlich HE, Wagner C, HilleRisLambers J, Tewksbury J, Harsch MA, Parrish JK (2015) Global change and local solutions: Tapping the unrealized potential of citizen science for biodiversity research. Biological Conservation 181, 236–244.

[r71] Vale MM, Tourinho L, Lorini ML, Rajão H and Figueiredo MdSL (2018) Endemic birds of the Atlantic Forest: traits, conservation status, and patterns of biodiversity. Journal of Field Ornithology 89(3), 193–206.

[r72] Wickham H (2016) ggplot2: Elegant Graphics for Data Analysis. New York: Springer-Verlag.

[r73] Wickham H, François R, Henry L and Müller K (2022) dplyr: a grammar of data manipulation. R package version 1.0.9. https://CRAN.R-project.org/package=dplyr (accessed 20 January 2023).

[r74] Zulian V, Miller DAW and Ferraz G (2021) Integrating citizen-science and planned-survey data improves species distribution estimates. Diversity and Distributions 27, 2498–2509.

