# Supplementary material for: Global threat status, rarity, and species distribution affect prevalence of Atlantic Forest endemic birds in citizen-collected datasets
Source: Camb Prism Extinct. 2024 Nov 22;2:e17. doi: 10.1017/ext.2024.22 (PMC11895707; doi:10.1017/ext.2024.22)
Supplement: Forti et al. supplementary material [file S2755095824000226sup001.zip › Supplementary Table3 new.docx]

**Supplementary Table 3.** The results of the Spearman correlations between log10-transformed extent of occurrence (EOO), distribution range (d) and the estimate of median abundance (μ) of endemic bird species of the Atlantic Forest. Although all correlations were significant, exact p-values could not be calculated.

|  | **log(EOO)** | **log(μ)** | **log(AOO)** |
| --- | --- | --- | --- |
| **log(EOO)** | 1 | 0.4772518 | 0.9089717 |
| **log(μ)** | - | 1 | 0.5307291 |
| **log(d)** | - | - | 1 |
