# Supplementary material for: Global threat status, rarity, and species distribution affect prevalence of Atlantic Forest endemic birds in citizen-collected datasets
Source: Camb Prism Extinct. 2024 Nov 22;2:e17. doi: 10.1017/ext.2024.22 (PMC11895707; doi:10.1017/ext.2024.22)
Supplement: Forti et al. supplementary material [file S2755095824000226sup001.zip › Supplementary Table2 new.docx]

**Supplementary Table 2.** Results of a generalised linear model (r^2^ = 0.5288) predicting the number of observations of 214 endemic Atlantic Forest birds (logarithmic value) in three citizen science platforms with interaction between the logarithm of the distribution range and threat status of species, SE – standard error, iucnEN, iucnLC, iucnNT, iucnVU are global threat categories based on IUCN (2022). * indicates significance at 0.05 and *** at 0.001 levels

| Variables | Estimate | SE | t-value | p-value |
| --- | --- | --- | --- | --- |
| (Intercept) | 1.3415 | 0.3663 | 3.662 | 0.00032*** |
| log10(range) | 0.2949 | 0.1315 | 2.243 | 0.02597* |
| iucnEN | 0.5455 | 0.5698 | 0.957 | 0.33954 |
| iucnLC | 0.8399 | 0.5687 | 1.477 | 0.14124 |
| iucnNT | 0.6201 | 0.6209 | 0.999 | 0.31911 |
| iucnVU | 1.2893 | 0.5079 | 2.538 | 0.01188* |
| log10(range):iucnEN | –0.1294 | 0.1721 | –0.752 | 0.45276 |
| log10(range):iucnLC | –0.0542 | 0.1521 | –0.356 | 0.72201 |
| log10(range):iucnNT | –0.0546 | 0.1627 | –0.335 | 0.73773 |
| log10(range):iucnVU | –0.2556 | 0.1538 | –1.662 | 0.09803 |
