# Supplementary material for: Global threat status, rarity, and species distribution affect prevalence of Atlantic Forest endemic birds in citizen-collected datasets
Source: Camb Prism Extinct. 2024 Nov 22;2:e17. doi: 10.1017/ext.2024.22 (PMC11895707; doi:10.1017/ext.2024.22)
Supplement: Forti et al. supplementary material [file S2755095824000226sup001.zip › Supplementary Table 1.docx]

**Supplementary Table 1.** Endemic bird species of the Brazilian Atlantic Forest based on Vale et al. (2018), with global threat status (IUCN 2022) and the number of observations (N) obtained from three digital citizen science platforms based on data from 2000–2022; LC – Least Concern, NT – Near Threatened, VU – Vulnerable, EN – Endangered, CR – Critically Endangered, EW – Extinct in the Wild, EX – Extinct. Min-max values for the population are marked in bold for **declining** species (165 species), italics for *stable*, increasing are underlined, and populations with unknown trend (11 species) are without marking (BirdLife International, 2023). Extent of Occurrence (EOO) was obtained from BirdLife International (2023). Distribution range (EOD), feeding behavior (trophic level) categories (invertivore: I, frugivore: F, granivore: G, nectarivore: N, omnivore: O, vertivore: V) and life history (aerial: a, generalist: g, insessorial: i, terrestrial: t) are based on data from Tobias et al. (2022). Res indicates the calculated residual values of the main model and N is the number of observations on citizen science platforms in 2000–2022. Note that we use BirdLife International taxonomy and nomenclature

| family and *species* | Status | min-max | EOO (km^2^) | EOD (km^2^) | Body mass (g) | Trophic level, life history | N | res |
| --- | --- | --- | --- | --- | --- | --- | --- | --- |
| Accipitridae |  |  |  |  |  |  |  |  |
| *Leptodon forbesi* | EN | **250-999** | 90100 | 52831.24 | 577 | O,i | 333 | -0.276 |
| *Pseudastur polionotus* | NT | **2500-9999** | 1500000 | 813060.78 | 703.7 | V,i | 1720 | -0.152 |
| *Buteogallus lacernulatus* | VU | **2500-9999** | 1040000 | 139998.95 | 312 | V,i | 1841 | 0.300 |
| Bucconidae |  |  |  |  |  |  |  |  |
| *Malacoptila striata* | LC | **72000-129000** | 1090000 | 728993.44 | 44.1 | I,i | 6572 | 0.267 |
| *Notharchus swainsoni* | LC | *NA* | 988000 | 927376.95 | 73.8 | I,i | 1365 | -0.453 |
| Caprimulgidae |  |  |  |  |  |  |  |  |
| *Macropsalis forcipata* | LC | **NA** | 871000 | 517277.53 | 139 | I,g | 844 | -0.577 |
| Conopophagidae |  |  |  |  |  |  |  |  |
| *Conopophaga melanops* | LC | *NA* | 83699 | 242500.41 | 20.1 | I,i | 6585 | 0.348 |
| *Conopophaga cearae* | NT | **1500-3200** | 449000 | 58582.69 | 22 | I,i | 710 | -0.227 |
| Corvidae |  |  |  |  |  |  |  |  |
| *Cyanocorax coeruleus* | NT | **NA** | 638000 | 521253.94 | 272 | O,i | 7582 | 0.479 |
| Cotingidae |  |  |  |  |  |  |  |  |
| *Cotinga maculata* | CR | **50-249** | 51800 | 30327.88 | 65 | F,i | 257 | -0.165 |
| *Carpornis cucullata* | LC | **NA** | 384000 | 251722.97 | 74.19 | F,i | 4600 | 0.156 |
| *Lipaugus ater* | LC | **20000-49999** | 38400 | 12392.12 | 80 | F,i | 1642 | -0.027 |
| *Lipaugus lanioides* | LC | **50000-99999** | 609000 | 201153.3 | 94.8 | F,i | 1669 | -0.273 |
| *Phibalura flavirostris* | LC | **20000-49999** | 1690000 | 1084489.75 | 46.5 | F,i | 1715 | -0.388 |
| *Carpornis melanocephala* | NT | **2500-9999** | 560000 | 67888.43 | 64.19 | F,i | 1223 | -0.030 |
| *Procnias nudicollis* | NT | **80000-130000** | 2100000 | 1467436.56 | 172.04 | F,i | 7788 | 0.470 |
| *Lipaugus conditus* | VU | *250-9999* | 1200 | 250.46 | 80 | F,i | 262 | 0.032 |
| *Xipholena atropurpurea* | VU | **2500-9999** | 250000 | 1836.59 | 61.32 | F,i | 701 | 0.291 |
| Cracidae |  |  |  |  |  |  |  |  |
| *Crax blumenbachii* | EN | **130-170** | 77400 | 1149.72 | 3500 | F,t | 554 | 0.194 |
| *Pipile jacutinga* | EN | **1500-7000** | 865000 | 169256.82 | 1240.96 | F,i | 1408 | 0.188 |
| *Ortalis araucuan* | LC | **NA** | 524000 | 349725.59 | 547.72 | F,i | 1971 | -0.313 |
| *Ortalis squamata* | LC | **NA** | 489000 | 290989.61 | 547.72 | F,i | 4437 | 0.056 |
| Formicariidae |  |  |  |  |  |  |  |  |
| *Chamaeza meruloides* | LC | *NA* | 541000 | 103365.42 | 69 | I,t | 2424 | 0.001 |
| Formicariidae |  |  |  |  |  |  |  |  |
| *Chamaeza ruficauda* | LC | **NA** | 644000 | 371454.37 | 71.2 | I,t | 1780 | -0.247 |
| Fringillidae |  |  |  |  |  |  |  |  |
| *Euphonia pectoralis* | LC | **NA** | 3350000 | 1361244.46 | 14.4 | F,i | 14946 | 0.499 |
| *Euphonia chalybea* | NT | **NA** | 999000 | 745412.86 | 19 | F,i | 2811 | 0.086 |
| Furnariidae |  |  |  |  |  |  |  |  |
| *Automolus lammi* | EN | **1000-2499** | 80600 | 63962.64 | 34.5 | I,i | 230 | -0.352 |
| *Synallaxis infuscata* | EN | **250-999** | 31100 | 752.18 | 18.2 | I,i | 109 | -0.261 |
| *Anabacerthia lichtensteini* | LC | **NA** | 2080000 | 1589284.85 | 21 | I,i | 2804 | -0.144 |
| *Anabazenops fuscus* | LC | **NA** | 629000 | 372722.52 | 38.5 | I,i | 3976 | 0.117 |
| *Asthenes moreirae* | LC | **NA** | 54800 | 39562.85 | 10.48 | I,i | 1031 | -0.229 |
| *Automolus leucophthalmus* | LC | **NA** | 2610000 | 1800565.07 | 34.5 | I,i | 8375 | 0.304 |
| *Campylorhamphus falcularius* | LC | **NA** | 1690000 | 876884.84 | 42.6 | I,i | 2579 | -0.150 |
| *Cichlocolaptes leucophrus* | LC | **NA** | 255000 | 148328.51 | 50 | I,i | 2710 | 0.024 |
| *Clibanornis dendrocolaptoides* | LC | **20000-49999** | 325000 | 452976.71 | 48.2 | I,t | 855 | -0.575 |
| *Cranioleuca obsoleta* | LC | **NA** | 693000 | 653975.04 | 13.4 | I,i | 3288 | 0.018 |
| *Cranioleuca pallida* | LC | **NA** | 918000 | 694100.17 | 11.5 | I,i | 10153 | 0.507 |
| *Dendrocincla turdina* | LC | **NA** | 1810000 | 1193430.6 | 39 | I,i | 6540 | 0.230 |
| *Heliobletus contaminatus* | LC | **NA** | 1100000 | 815159.11 | 14 | I,i | 1512 | -0.340 |
| *Lepidocolaptes falcinellus* | LC | *NA* | 650000 | 497665.62 | 27.92 | I,i | 4479 | 0.153 |
| *Lepidocolaptes squamatus* | LC | **NA** | 1140000 | 745438.76 | 28.1 | I,i | 3940 | 0.062 |
| *Leptasthenura striolata* | LC | *NA* | 214000 | 208971.09 | 10.5 | I,i | 993 | -0.393 |
| *Phacellodomus erythrophthalmus* | LC | **NA** | 508000 | 394570.11 | 24.5 | I,i | 4006 | 0.130 |
| *Phacellodomus ferrugineigula* | LC | *NA* | 905000 | 586137.08 | 24.5 | I,i | 3410 | 0.025 |
| *Philydor atricapillus* | LC | **NA** | 1720000 | 1129776.92 | 22.2 | I,i | 5525 | 0.179 |
| *Sclerurus scansor* | LC | **NA** | 2560000 | 2117654.84 | 36.9 | I,t | 4279 | -0.004 |
| *Synallaxis ruficapilla* | LC | *NA* | 1330000 | 1174516.01 | 13.8 | I,i | 13720 | 0.586 |
| *Xiphorhynchus fuscus* | LC | **NA** | 2450000 | 1948346.79 | 21.8 | I,i | 10401 | 0.406 |
| *Anabacerthia amaurotis* | NT | **NA** | 724000 | 499233.47 | 19.2 | I,i | 1539 | -0.030 |
| *Cinclodes pabsti* | NT | **NA** | 125000 | 21292.38 | 53 | I,t | 1275 | 0.136 |
| *Leptasthenura setaria* | NT | **NA** | 501000 | 394948.9 | 11 | I,i | 4866 | 0.508 |
| *Synallaxis cinerea* | NT | **4400-13200** | 89800 | 24736.24 | 16.2 | I,i | 448 | -0.294 |
| *Acrobatornis fonsecai* | VU | **2500-9999** | 13400 | 3689.54 | 13.7 | I,i | 234 | -0.161 |
| *Sclerurus cearensis* | VU | **NA** | 658000 | 635484.22 | 31 | I,t | 165 | -0.796 |
| *Thripophaga macroura* | VU | **1500-7000** | 112000 | 5966.24 | 27.7 | I,i | 583 | 0.170 |
| *Xiphorhynchus atlanticus* | VU | **5000-11000** | 266000 | 326684.31 | 21.8 | I,i | 790 | -0.046 |
| Galbulidae |  |  |  |  |  |  |  |  |
| *Jacamaralcyon tridactyla* | NT | **1300-5400** | 253000 | 389660.14 | 18.3 | I,i | 1161 | -0.140 |
| Grallariidae |  |  |  |  |  |  |  |  |
| *Hylopezus nattereri* | LC | *NA* | 642000 | 493987 | 32 | I,t | 681 | -0.614 |
| Icteridae |  |  |  |  |  |  |  |  |
| *Anumara forbesi* | VU | **600-10000** | 447000 | 112.34 | 65.9 | I,g | 288 | 0.140 |
| Mitrospingidae |  |  |  |  |  |  |  |  |
| *Orthogonys chloricterus* | LC | **NA** | 302000 | 121968.52 | 39 | I,i | 5418 | 0.282 |
| Momotidae |  |  |  |  |  |  |  |  |
| *Baryphthengus ruficapillus* | LC | **NA** | 1960000 | 1565635.85 | 141.65 | I,i | 5809 | 0.073 |
| Odontophoridae |  |  |  |  |  |  |  |  |
| *Odontophorus capueira* | LC | **NA** | 3170000 | 1724256.7 | 425.4 | O,t | 3168 | -0.197 |
| Passerellidae |  |  |  |  |  |  |  |  |
| *Arremon semitorquatus* | LC | **NA** | 472000 | 215137.21 | 25 | O,i | 2873 | 0.008 |
| Picidae |  |  |  |  |  |  |  |  |
| *Campephilus robustus* | LC | NA | 2070000 | 1885815.7 | 200 | I,i | 4911 | 0.151 |
| *Melanerpes flavifrons* | LC | NA | 2220000 | 1557771.44 | 57.78 | O,i | 9249 | 0.482 |
| *Piculus chrysochloros* | LC | **500000-4999999** | 13400000 | 8623251.86 | 88 | I,i | 59 | -1.879 |
| *Picumnus exilis* | LC | **NA** | 5030000 | 1241697.36 | 9.3 | I,i | 1273 | -0.301 |
| *Picumnus temminckii* | LC | NA | 648000 | 514581.53 | 11.5 | I,i | 10963 | 0.705 |
| *Veniliornis maculifrons* | LC | *NA* | 370000 | 253876.59 | 38.69 | I,i | 1703 | -0.079 |
| *Piculus aurulentus* | NT | **NA** | 1120000 | 748842.17 | 74.99 | I,i | 5092 | 0.548 |
| *Celeus galeatus* | VU | **700-3600** | 412000 | 183768.38 | 124 | I,i | 335 | -0.284 |
| *Celeus tinnunculus* | VU | **NA** | 146000 | 120305.45 | 134 | I,i | 159 | -0.573 |
| Pipridae |  |  |  |  |  |  |  |  |
| *Antilophia bokermanni* | CR | **150-700** | 300 | 24.23 | 19.8 | F,i | 7 | -0.998 |
| *Chiroxiphia caudata* | LC | *NA* | 1920000 | 1459549.86 | 25.6 | O,i | 26382 | 0.852 |
| *Ilicura militaris* | LC | **NA** | 1160000 | 840759.23 | 12.7 | F,i | 7292 | 0.365 |
| *Machaeropterus regulus* | LC | **NA** | 241000 | 125343.35 | 9.34 | F,i | 997 | -0.320 |
| *Neopelma chrysolophum* | LC | *NA* | 194000 | 159645.19 | 14.2 | F,i | 2188 | -0.014 |
| *Neopelma aurifrons* | NT | **NA** | 142000 | 5071.66 | 14 | F,i | 327 | -0.264 |
| Polioptilidae |  |  |  |  |  |  |  |  |
| *Polioptila lactea* | NT | **NA** | 648000 | 441184.79 | 6.5 | I,i | 331 | -0.600 |
| Psittacidae |  |  |  |  |  |  |  |  |
| *Amazona vinacea* | EN | **1000-2499** | 1230000 | 105992.85 | 254 | O,i | 2863 | 0.464 |
| *Pyrrhura griseipectus* | EN | 250-2499 | 3300 | 686.2 | 75.9 | O,i | 635 | 0.296 |
| *Brotogeris tirica* | LC | *NA* | 1010000 | 288572.86 | 63 | O,i | 34619 | 0.893 |
| *Pionopsitta pileata* | LC | **NA** | 1650000 | 728878.23 | 119 | O,i | 4045 | -0.142 |
| *Pyrrhura frontalis* | LC | *NA* | 2690000 | 1704534.47 | 72 | O,i | 24417 | 0.579 |
| *Triclaria malachitacea* | LC | **10000-19999** | 377000 | 30242.84 | 90 | O,i | 1533 | -0.272 |
| *Amazona brasiliensis* | NT | 6000-6700 | 10100 | 4747.02 | 430 | O,i | 1204 | 0.007 |
| *Touit melanonotus* | NT | **2500-9999** | 537000 | 14296.54 | 66.51 | F,i | 640 | -0.307 |
| *Amazona pretrei* | VU | *15600-15600* | 161000 | 10426.1 | 272.76 | O,i | 882 | 0.057 |
| *Amazona rhodocorytha* | VU | **2500-9999** | 168000 | 2672.56 | 474.34 | O,i | 1313 | 0.333 |
| *Pionus reichenowi* | VU | **2500-9999** | 243000 | 133893.03 | 251 | O,i | 267 | -0.687 |
| *Pyrrhura cruentata* | VU | **2500-9999** | 281000 | 10274.69 | 75.9 | O,i | 887 | 0.101 |
| *Pyrrhura leucotis* | VU | **2500-9999** | 352000 | 273322.17 | 75.9 | O,i | 899 | -0.185 |
| *Touit surdus* | VU | **2500-9999** | 1680000 | 98992.48 | 51 | F,i | 860 | -0.101 |
| Rallidae |  |  |  |  |  |  |  |  |
| *Aramides saracura* | LC | **NA** | 1380000 | 928675.91 | 540 | O,t | 21249 | 0.572 |
| Ramphastidae |  |  |  |  |  |  |  |  |
| *Ramphastos dicolorus* | LC | **NA** | 1730000 | 1394133.06 | 331 | F,i | 17762 | 0.509 |
| *Selenidera maculirostris* | LC | **NA** | 1370000 | 930797.49 | 164 | F,i | 4223 | -0.057 |
| *Pteroglossus bailloni* | NT | **NA** | 1630000 | 657171.58 | 146 | F,i | 1755 | -0.135 |
| Rhinocryptidae |  |  |  |  |  |  |  |  |
| *Merulaxis stresemanni* | CR | **1-49** | 34 | 21.75 | 35 | I,t | 57 | 0.088 |
| *Eleoscytalopus psychopompus* | EN | **250-999** | 4185 | 5194.76 | 17.8 | I,t | 127 | -0.161 |
| *Scytalopus diamantinensis* | EN | **2500-9999** | 3380 | 21167.42 | 16.4 | I,t | 105 | -0.365 |
| *Scytalopus gonzagai* | EN | **1000-2499** | 3300 | 4360.15 | 13 | I,t | 9 | -1.285 |
| *Scytalopus iraiensis* | EN | **250-999** | 381000 | 5149.75 | 12.4 | I,g | 198 | 0.045 |
| *Eleoscytalopus indigoticus* | LC | **NA** | 1048000 | 454702.62 | 16.15 | I,t | 3571 | 0.285 |
| *Merulaxis ater* | LC | **NA** | 267000 | 163731.48 | 35.03 | I,t | 2188 | 0.139 |
| *Psilorhamphus guttatus* | LC | **NA** | 712000 | 410870.11 | 11.3 | I,i | 1535 | -0.061 |
| *Scytalopus pachecoi* | LC | *NA* | 259000 | 46604.22 | 15 | I,g | 510 | -0.355 |
| *Scytalopus petrophilus* | LC | *NA* | 90000 | 46263.72 | 13.5 | I,g | 537 | -0.329 |
| *Scytalopus speluncae* | LC | **NA** | 400000 | 249767.52 | 13 | I,t | 3057 | 0.278 |
| Strigidae |  |  |  |  |  |  |  |  |
| *Glaucidium minutissimum* | LC | **NA** | 1460000 | 1209018.27 | 50 | I,i | 1735 | -0.252 |
| *Megascops atricapilla* | LC | *NA* | 2180000 | 990012.51 | 118.89 | I,i | 1521 | -0.319 |
| *Megascops sanctaecatarinae* | LC | **NA** | 426000 | 418281.17 | 179.72 | I,i | 1296 | -0.325 |
| *Pulsatrix koeniswaldiana* | LC | **NA** | 1040000 | 651781.62 | 481 | V,i | 3827 | 0.075 |
| *Strix hylophila* | LC | **NA** | 1200000 | 711556.62 | 345.38 | V,i | 2202 | -0.162 |
| Thamnophilidae |  |  |  |  |  |  |  |  |
| *Formicivora paludicola* | CR | 150-700 | 1500 | 487.85 | 9.3 | I,i | 350 | 0.307 |
| *Myrmotherula snowi* | CR | **1-49** | 230 | 197.95 | 9.59 | I,i | 102 | -0.149 |
| *Terenura sicki* | CR | **50-249** | 14600 | 132.4 | 6.4 | I,i | 289 | 0.352 |
| *Formicivora erythronotos* | EN | **50-8500** | 410 | 131.33 | 10.3 | I,i | 488 | 0.434 |
| *Myrmoderus ruficauda* | EN | **600-1700** | 155000 | 499.32 | 26 | I,i | 571 | 0.354 |
| *Pyriglena atra* | EN | **600-1700** | 9200 | 4969.97 | 32 | I,i | 243 | -0.228 |
| *Rhopornis ardesiacus* | EN | **600-1700** | 12000 | 2437.29 | 26.3 | I,i | 528 | 0.179 |
| *Drymophila ferruginea* | LC | *NA* | 965000 | 506171.48 | 10.6 | I,i | 7324 | 0.267 |
| *Drymophila genei* | LC | **6000-58400** | 114000 | 24959.28 | 11.4 | I,i | 1667 | -0.111 |
| *Drymophila malura* | LC | *NA* | 1100000 | 769759.91 | 13 | I,i | 3715 | -0.072 |
| *Drymophila rubricollis* | LC | *NA* | 437000 | 329402.94 | 10 | I,i | 2242 | -0.207 |
| *Drymophila squamata* | LC | *NA* | 984000 | 340038.68 | 10.8 | I,i | 5064 | 0.141 |
| *Dysithamnus xanthopterus* | LC | **NA** | 188000 | 44440.17 | 16 | I,i | 1434 | -0.238 |
| *Formicivora serrana* | LC | **NA** | 267000 | 209781.99 | 11.4 | I,i | 1487 | -0.350 |
| *Hypoedaleus guttatus* | LC | **NA** | 2210000 | 829945.45 | 38.8 | I,i | 6559 | 0.134 |
| *Mackenziaena leachii* | LC | **NA** | 1150000 | 828525.05 | 70.2 | I,i | 3546 | -0.152 |
| *Mackenziaena severa* | LC | *NA* | 1390000 | 955402.98 | 51.8 | I,i | 4471 | -0.054 |
| *Myrmoderus loricatus* | LC | **NA** | 404000 | 177950.43 | 15.5 | I,g | 1720 | -0.282 |
| *Myrmoderus squamosus* | LC | **NA** | 445000 | 241730.2 | 18.5 | I,g | 5380 | 0.181 |
| *Myrmotherula axillaris* | LC | **NA** | 10400000 | 7734412.17 | 8.09 | I,i | 1213 | -0.748 |
| *Pyriglena leucoptera* | LC | **NA** | 2010000 | 1228571.5 | 28.8 | I,i | 11675 | 0.359 |
| *Rhopias gularis* | LC | **NA** | 847000 | 500928.79 | 11.3 | I,i | 5203 | 0.117 |
| *Terenura maculata* | LC | **NA** | 1340000 | 783959.13 | 6.5 | I,i | 4982 | 0.076 |
| *Thamnophilus ambiguus* | LC | *NA* | 342000 | 209875.65 | 23.4 | I,i | 1873 | -0.272 |
| *Cercomacra brasiliana* | NT | **10000-19999** | 266000 | 129178.51 | 12 | I,i | 598 | -0.435 |
| *Drymophila ochropyga* | NT | **NA** | 1050000 | 359922.99 | 10.5 | I,i | 1879 | -0.025 |
| *Dysithamnus stictothorax* | NT | **NA** | 889000 | 415220.51 | 15.5 | I,i | 3938 | 0.272 |
| *Formicivora acutirostris* | NT | **5000-9999** | 27900 | 880.77 | 10 | I,i | 898 | 0.190 |
| *Myrmotherula unicolor* | NT | **10000-19999** | 237000 | 69111.62 | 11.5 | I,i | 3364 | 0.372 |
| *Biatas nigropectus* | VU | **2500-9999** | 605000 | 23893.22 | 25.5 | I,i | 1245 | 0.250 |
| *Dysithamnus plumbeus* | VU | **2500-15000** | 141000 | 42463.62 | 20.5 | I,i | 353 | -0.342 |
| *Herpsilochmus pileatus* | VU | **1000-2499** | 23400 | 1545.44 | 8.9 | I,i | 624 | 0.226 |
| *Myrmotherula minor* | VU | **2500-9999** | 189000 | 4980.73 | 6.4 | I,i | 659 | 0.157 |
| *Myrmotherula urosticta* | VU | **1500-7000** | 119000 | 3033.06 | 8.4 | I,i | 446 | 0.023 |
| Thraupidae |  |  |  |  |  |  |  |  |
| *Nemosia rourei* | CR | **30-200** | 2200 | 52.79 | 22 | I,i | 147 | 0.021 |
| *Castanozoster thoracicus* | LC | **NA** | 273000 | 77555.65 | 11.9 | I,i | 1978 | -0.218 |
| *Haplospiza unicolor* | LC | **NA** | 1160000 | 977656.66 | 15.28 | G,g | 3948 | -0.151 |
| *Hemithraupis ruficapilla* | LC | *NA* | 949000 | 648694.04 | 11 | I,i | 10180 | 0.307 |
| *Microspingus lateralis* | LC | **NA** | 76400 | 57423.08 | 19.2 | O,i | 3726 | 0.069 |
| *Ramphocelus bresilius* | LC | *NA* | 1420000 | 377620.27 | 32.9 | O,i | 21613 | 0.648 |
| *Saltator fuliginosus* | LC | NA | 2550000 | 811739.38 | 44 | I,i | 4827 | -0.081 |
| *Saltator maxillosus* | LC | NA | 731000 | 436886.3 | 50.8 | Herbivore,i | 2935 | -0.246 |
| *Tachyphonus coronatus* | LC | *NA* | 1950000 | 1492450.62 | 29.3 | I,i | 35583 | 0.746 |
| *Tangara brasiliensis* | LC | **NA** | 419000 | 146051.89 | 20.5 | O,i | 827 | -0.670 |
| *Tangara cyanocephala* | LC | *NA* | 2610000 | 444844.92 | 18 | F,i | 13062 | 0.434 |
| *Tangara cyanomelas* | LC | **NA** | 392000 | 212091.26 | 21 | F,i | 465 | -0.954 |
| *Tangara cyanoventris* | LC | *NA* | 759000 | 475625.93 | 16.5 | F,i | 6928 | 0.155 |
| *Tangara desmaresti* | LC | *NA* | 445000 | 260465.02 | 20.4 | F,i | 8705 | 0.301 |
| *Tangara ornata* | LC | *NA* | 631000 | 392822.74 | 33 | F,i | 6297 | 0.109 |
| *Tangara seledon* | LC | *NA* | 1560000 | 672563.84 | 18.7 | F,i | 21755 | 0.617 |
| *Thlypopsis pyrrhocoma* | LC | *NA* | 842000 | 611568.56 | 15.6 | I,i | 2298 | -0.345 |
| *Dacnis nigripes* | NT | **6700-6700** | 250000 | 27779.66 | 14 | O,i | 1239 | -0.066 |
| *Orchesticus abeillei* | NT | **NA** | 387000 | 163525.7 | 31.5 | I,i | 1804 | -0.086 |
| *Tangara cyanoptera* | NT | **NA** | 919000 | 224069.57 | 43.3 | F,i | 3316 | 0.140 |
| *Sporophila falcirostris* | VU | **2500-9999** | 1200000 | 172403.15 | 13.5 | G,i | 1157 | -0.017 |
| *Sporophila frontalis* | VU | **2500-9999** | 1040000 | 186188.74 | 19.3 | G,i | 2049 | 0.213 |
| *Tangara fastuosa* | VU | **2500-9999** | 39400 | 15681.33 | 25.5 | F,i | 964 | 0.097 |
| *Tangara peruviana* | VU | **2500-9999** | 316000 | 56462.68 | 22.2 | F,i | 1912 | 0.285 |
| Tinamidae |  |  |  |  |  |  |  |  |
| *Tinamus solitarius* | NT | **50000-200000** | 1930000 | 1069608.94 | 1386.41 | O,t | 3327 | 0.063 |
| Tityridae |  |  |  |  |  |  |  |  |
| *Iodopleura pipra* | EN | **250-2499** | 767000 | 130157.72 | 10.03 | F,i | 1341 | 0.360 |
| *Schiffornis virescens* | LC | **NA** | 2070000 | 1662556.58 | 25.6 | O,i | 8586 | 0.302 |
| *Laniisoma elegans* | NT | **NA** | 506000 | 172527.96 | 47.4 | O,i | 330 | -0.663 |
| Trochilidae |  |  |  |  |  |  |  |  |
| *Thalurania watertonii* | EN | **1000-2499** | 39800 | 34673.09 | 4.6 | N,a | 716 | 0.197 |
| *Clytolaema rubricauda* | LC | NA | 642000 | 352748.43 | 7.9 | N,a | 4023 | 0.114 |
| *Phaethornis eurynome* | LC | **NA** | 1690000 | 934246.25 | 5.3 | N,a | 9546 | 0.416 |
| *Phaethornis idaliae* | LC | **NA** | 193000 | 154965.58 | 2.6 | N,a | 800 | -0.479 |
| *Phaethornis malaris* | LC | **NA** | 10300000 | 3790374.78 | 5.9 | N,a | 104 | -1.675 |
| *Phaethornis squalidus* | LC | NA | 652000 | 272378.96 | 3.4 | N,a | 1984 | -0.143 |
| *Ramphodon naevius* | LC | **175000-1710000** | 378000 | 220311.75 | 7.9 | N,a | 7657 | 0.436 |
| *Stephanoxis lalandi* | LC | NA | 336000 | 300448.07 | 4 | N,a | 2489 | -0.058 |
| *Stephanoxis loddigesii* | LC | NA | 893000 | 834254.78 | 4 | N,a | 2487 | -0.149 |
| *Thalurania glaucopis* | LC | NA | 2490000 | 1415794.42 | 4.8 | N,a | 30893 | 0.892 |
| *Lophornis chalybeus* | NT | NA | 332000 | 182459.44 | 3 | N,a | 3993 | 0.469 |
| *Glaucis dohrnii* | VU | **2500-9999** | 102000 | 978.16 | 5.8 | N,a | 468 | 0.222 |
| Trogonidae |  |  |  |  |  |  |  |  |
| *Trogon surrucura* | LC | **NA** | 1970000 | 1366931 | 73.29 | I,i | 8433 | 0.244 |
| Tyrannidae |  |  |  |  |  |  |  |  |
| *Phylloscartes ceciliae* | CR | **50-249** | 11800 | 18944.19 | 7.6 | I,i | 339 | 0.005 |
| *Phylloscartes beckeri* | EN | **2500-9999** | 1500 | 2304.78 | 8.7 | I,i | 237 | -0.098 |
| *Attila rufus* | LC | **NA** | 848000 | 342429.82 | 42.6 | I,i | 10287 | 0.436 |
| *Hemitriccus diops* | LC | **NA** | 1420000 | 737548.44 | 10 | I,i | 3138 | -0.102 |
| *Hemitriccus nidipendulus* | LC | **NA** | 949000 | 542148.01 | 7.5 | I,i | 4478 | 0.089 |
| *Hemitriccus obsoletus* | LC | **NA** | 464000 | 262970.93 | 11.6 | I,i | 1603 | -0.307 |
| *Knipolegus nigerrimus* | LC | *NA* | 2260000 | 789107.65 | 20.3 | I,i | 5281 | 0.096 |
| *Mionectes rufiventris* | LC | **NA** | 1280000 | 1057487.4 | 13.3 | I,i | 7262 | 0.221 |
| *Muscipipra vetula* | LC | *NA* | 1060000 | 828420 | 27 | I,i | 3475 | -0.100 |
| *Myiornis auricularis* | LC | **NA** | 2090000 | 1267042.85 | 5.3 | I,i | 7675 | 0.258 |
| *Phyllomyias virescens* | LC | **NA** | 1320000 | 1029199.71 | 8.2 | I,i | 1946 | -0.333 |
| *Phylloscartes difficilis* | LC | **20000-49999** | 288000 | 153868.01 | 6.9 | I,i | 700 | -0.603 |
| *Phylloscartes kronei* | LC | **2500-9999** | 58500 | 5479.39 | 8.3 | I,i | 1751 | 0.086 |
| *Todirostrum poliocephalum* | LC | *NA* | 726000 | 465051.92 | 5.6 | I,i | 18110 | 0.719 |
| *Hemitriccus orbitatus* | NT | **NA** | 1010000 | 663643.42 | 9.7 | I,i | 3256 | 0.193 |
| *Phyllomyias griseocapilla* | NT | **NA** | 596000 | 190093.2 | 8 | I,i | 4138 | 0.414 |
| *Phylloscartes oustaleti* | NT | **NA** | 219000 | 74884.82 | 10 | I,i | 1890 | 0.150 |
| *Phylloscartes paulista* | NT | **1500-7000** | 818000 | 473320.65 | 7.48 | I,i | 1441 | -0.123 |
| *Phylloscartes sylviolus* | NT | **NA** | 832000 | 434888.86 | 8 | I,i | 1001 | -0.276 |
| *Piprites pileata* | NT | **2800-22400** | 356000 | 45097.1 | 15 | F,i | 1312 | 0.023 |
| *Pogonotriccus eximius* | NT | **NA** | 1170000 | 1008457.47 | 7.5 | I,i | 893 | -0.398 |
| *Hemitriccus furcatus* | VU | **2500-9999** | 238000 | 7556.76 | 9.4 | I,i | 1431 | 0.475 |
| *Hemitriccus kaempferi* | VU | **6000-12000** | 11700 | 7847.61 | 8.5 | I,i | 683 | 0.154 |
| *Hemitriccus mirandae* | VU | **1500-7000** | 212000 | 71439.38 | 10 | I,i | 541 | -0.149 |
| *Onychorhynchus swainsoni* | VU | **600-1700** | 288000 | 38092.82 | 17 | I,i | 765 | 0.040 |
| *Platyrinchus leucoryphus* | VU | **2500-9999** | 852000 | 425471.32 | 16 | I,i | 487 | -0.368 |
| Vireonidae |  |  |  |  |  |  |  |  |
| *Hylophilus poicilotis* | LC | *NA* | 983000 | 673378.96 | 10.4 | I,i | 7960 | 0.332 |
