# Supplementary material for: Global threat status, rarity, and species distribution affect prevalence of Atlantic Forest endemic birds in citizen-collected datasets
Source: Camb Prism Extinct. 2024 Nov 22;2:e17. doi: 10.1017/ext.2024.22 (PMC11895707; doi:10.1017/ext.2024.22)
Supplement: Forti et al. supplementary material [file S2755095824000226sup001.zip › fig2_pdf.pdf]

Monitoring endemic
Atlantic Forest In Braz
citizen-science platfor
WikiAves, eBird and i

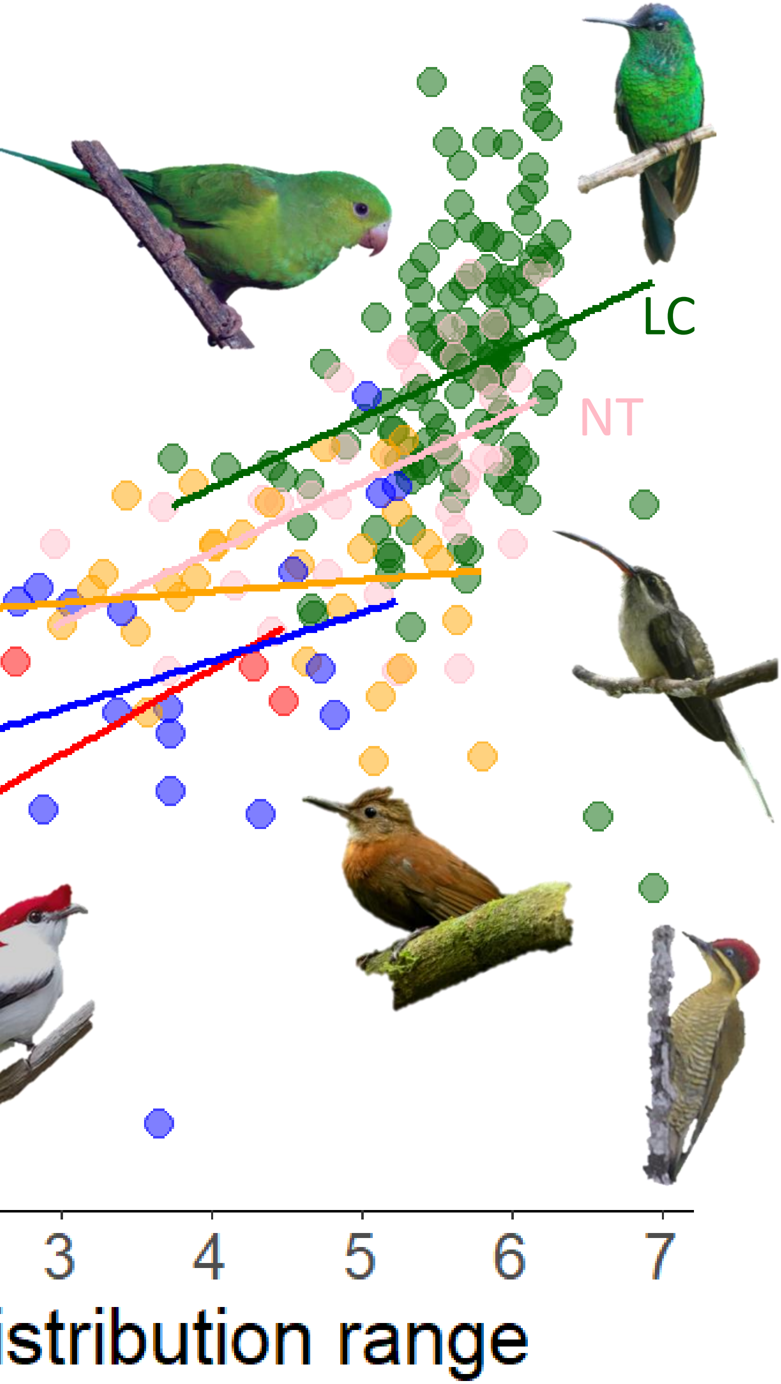

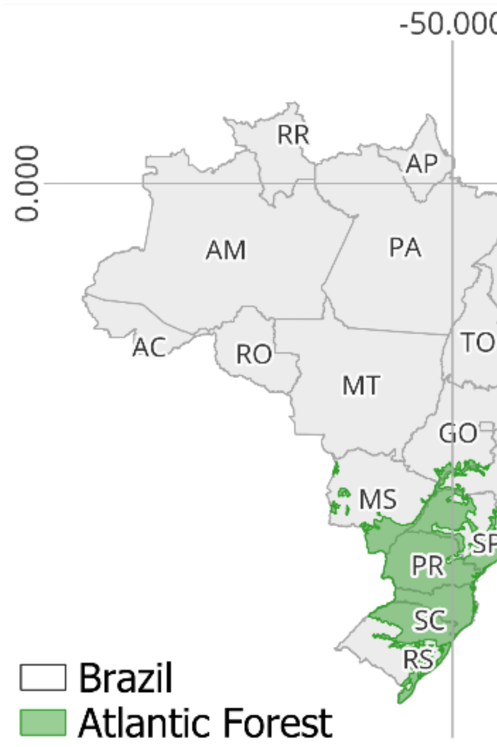

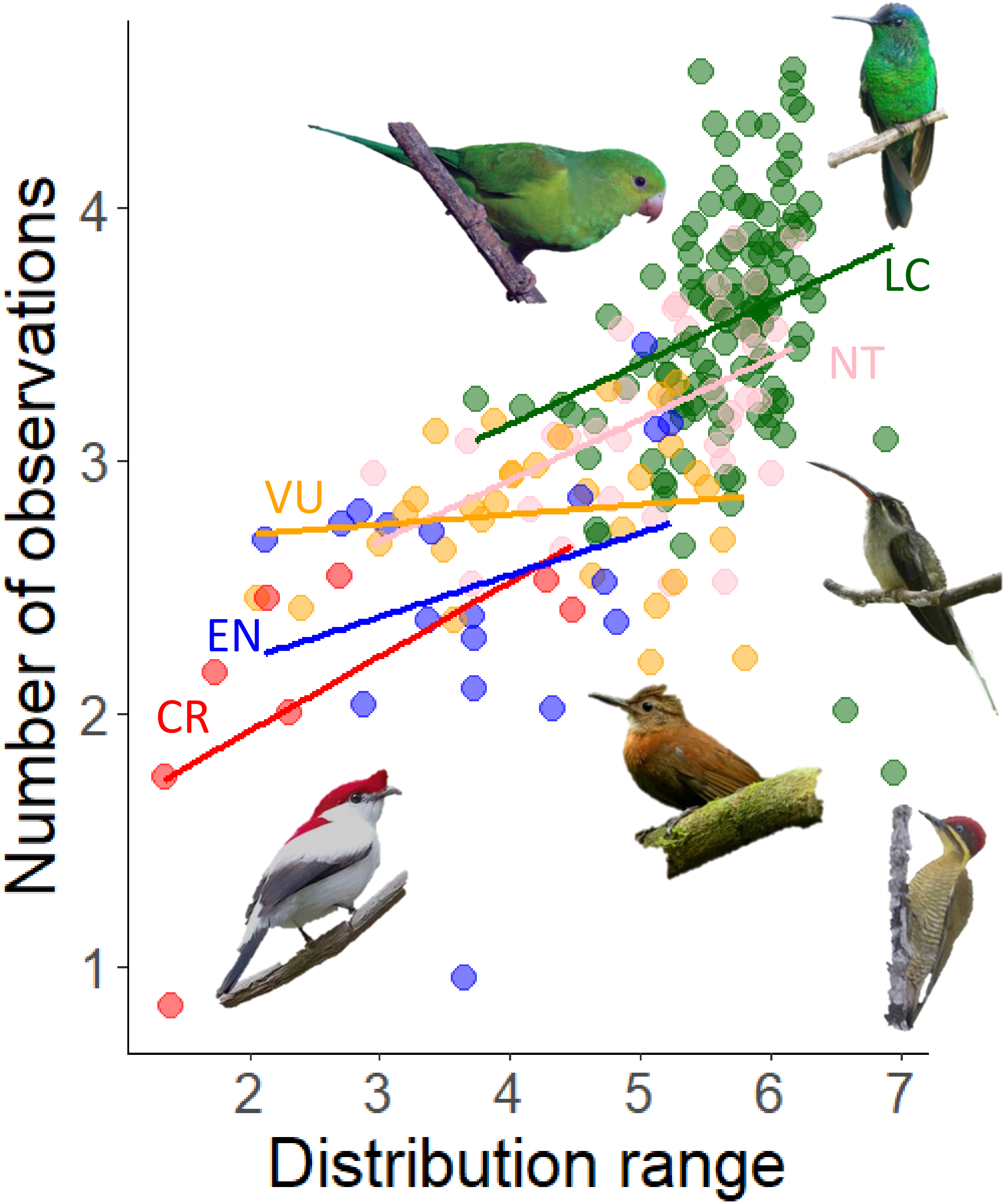
Number of observations
Distribution range
1
2
3
4
2
3
4
5
6
7
LC
NT
VU
EN
CR

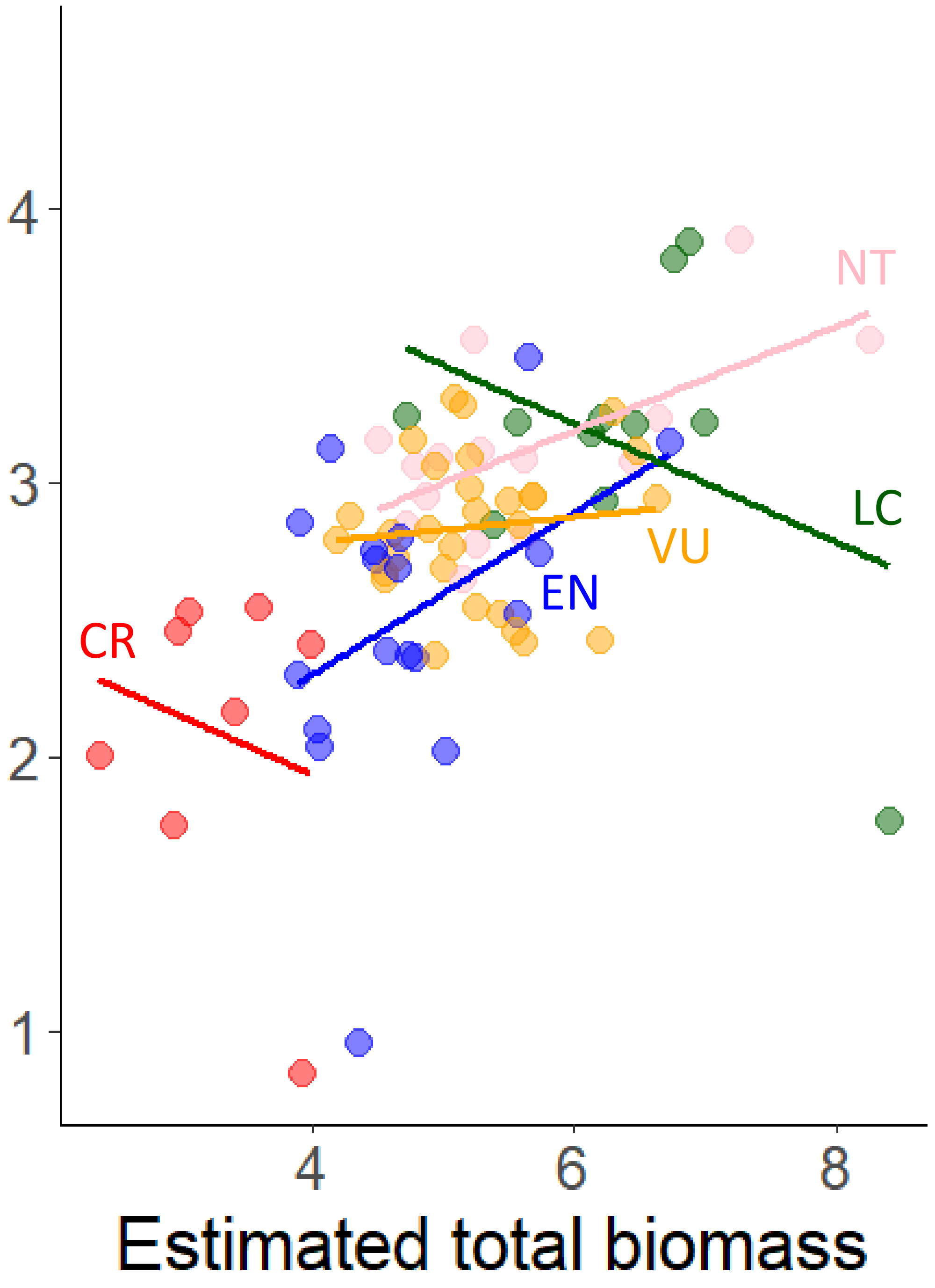

4
3
2
1
NT
LC
VU
EN
CR
4
6
8
Estimated total biomass

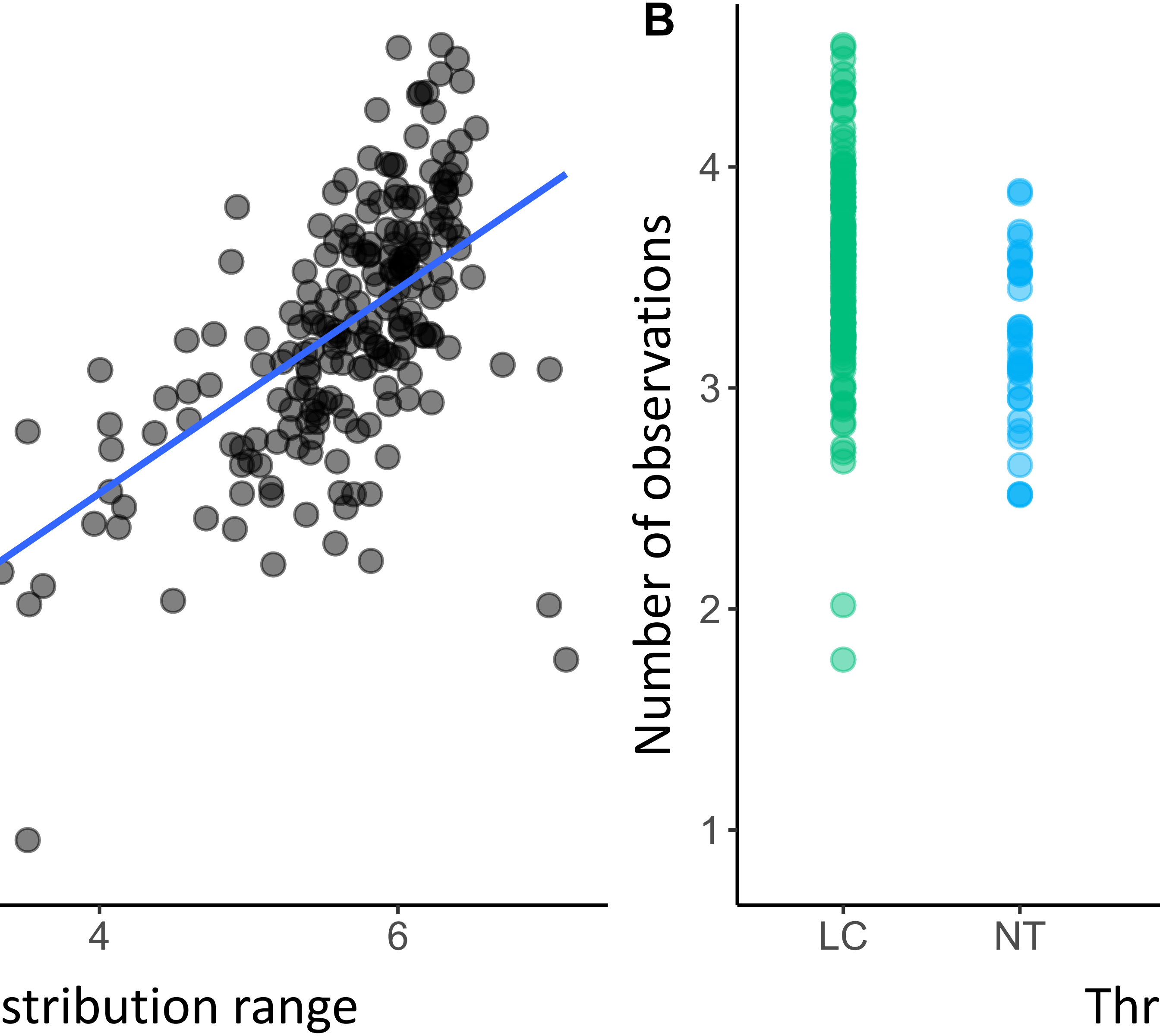

B
4
3
2
1
Number of observations
LC
NT
4
6
stribution range
Thr

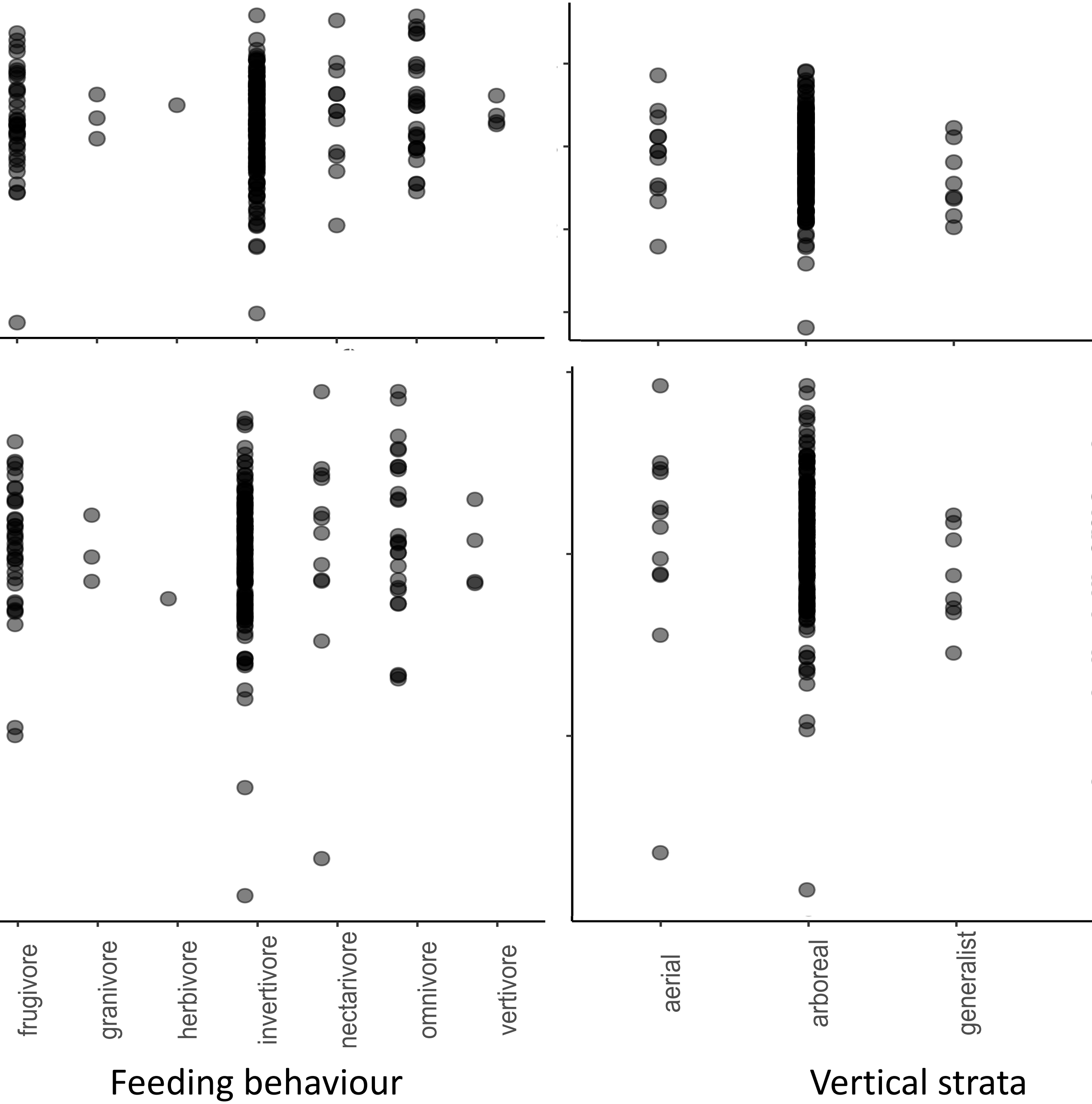

frugivore
granivore
herbivore
invertivore
nectarivore
omnivore
vertivore
Feeding behaviour
aerial
arboreal
generalist
Vertical strata
