# Supplementary figures and images for: Global threat status, rarity, and species distribution affect prevalence of Atlantic Forest endemic birds in citizen-collected datasets
Source: Camb Prism Extinct. 2024 Nov 22;2:e17. doi: 10.1017/ext.2024.22 (PMC11895707; doi:10.1017/ext.2024.22)

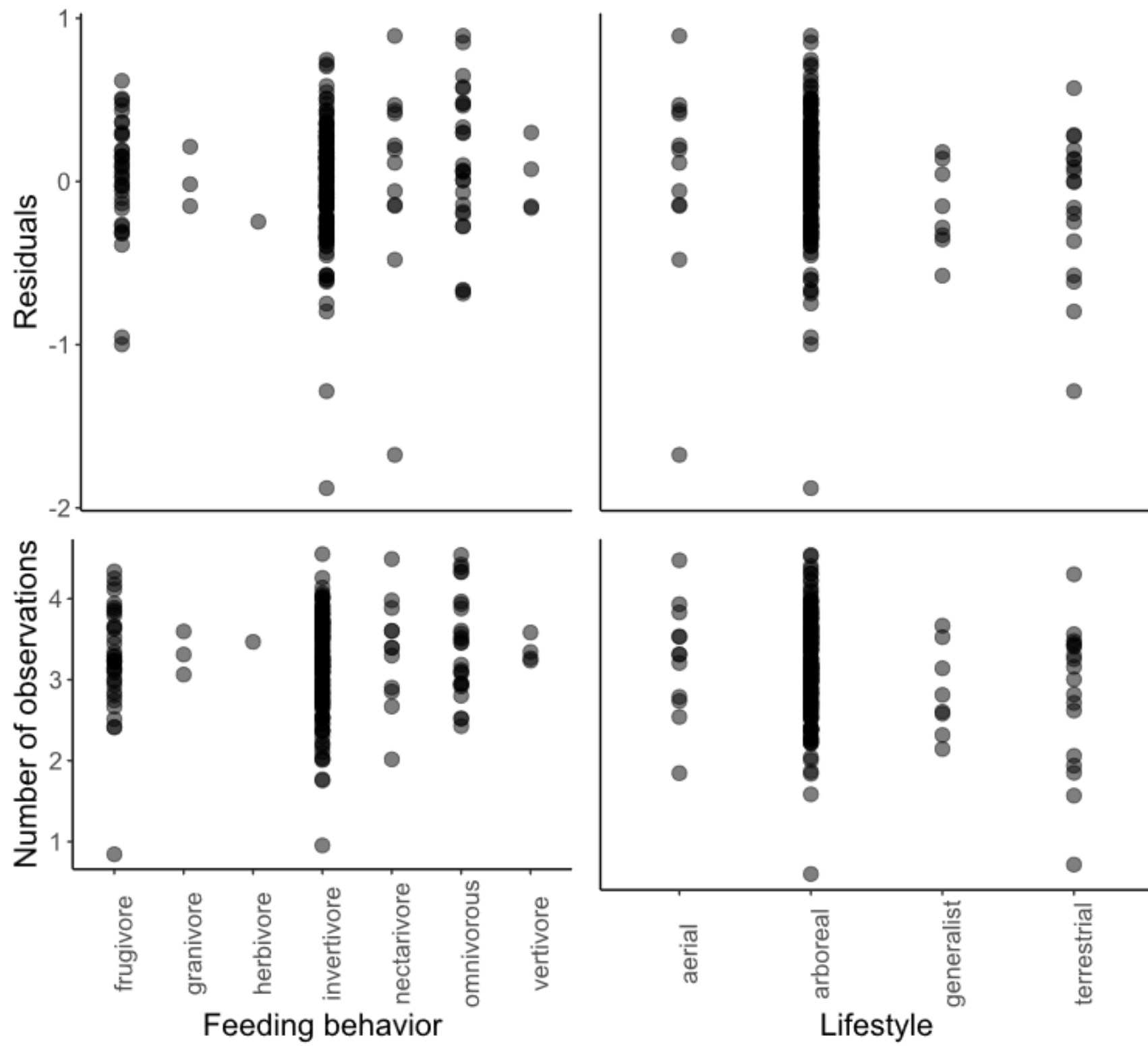

Supplement: Forti et al. supplementary material [file S2755095824000226sup001.zip › fig 4_new.pdf]

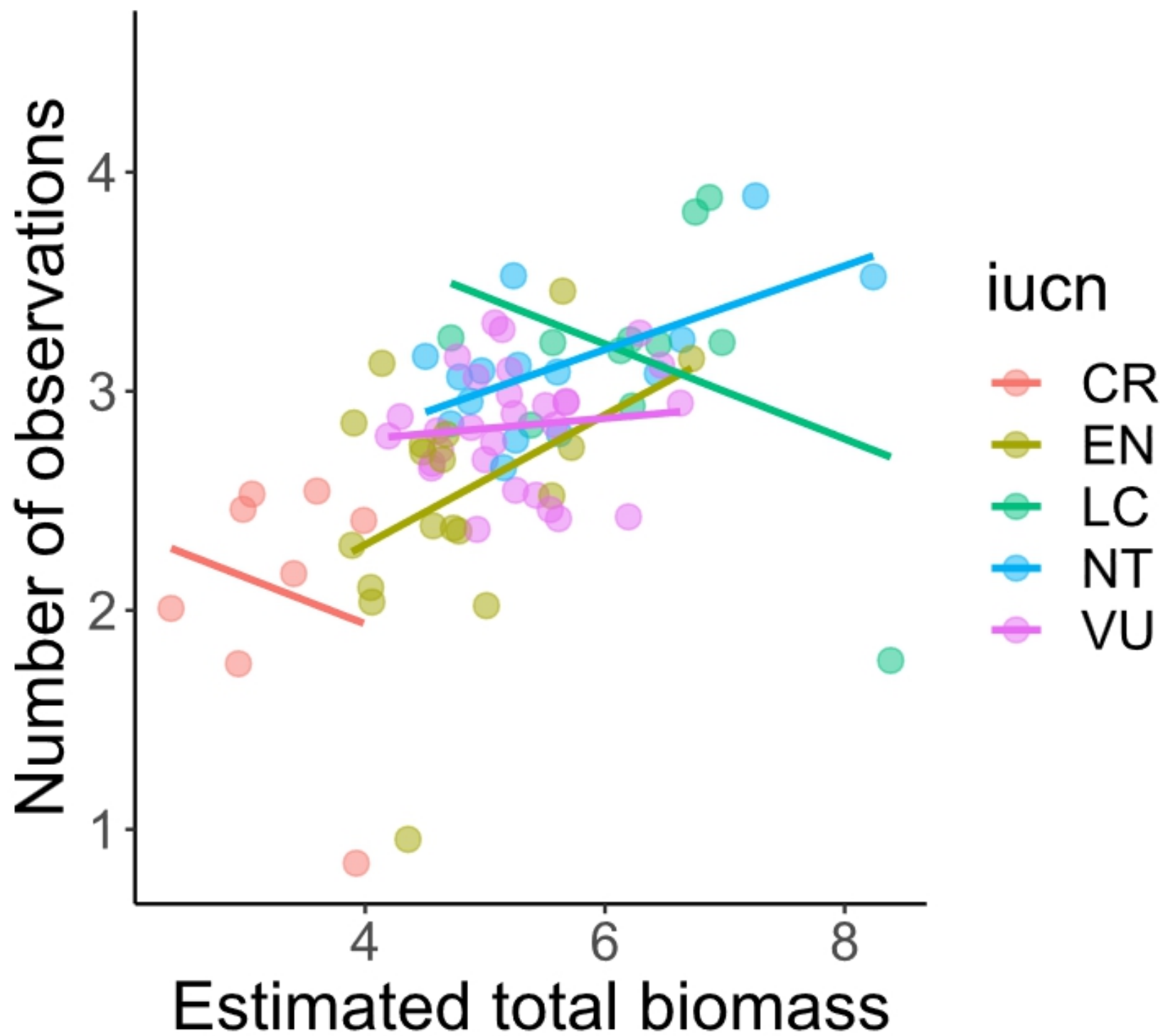

Supplement: Forti et al. supplementary material [file S2755095824000226sup001.zip › fig 3.pdf]

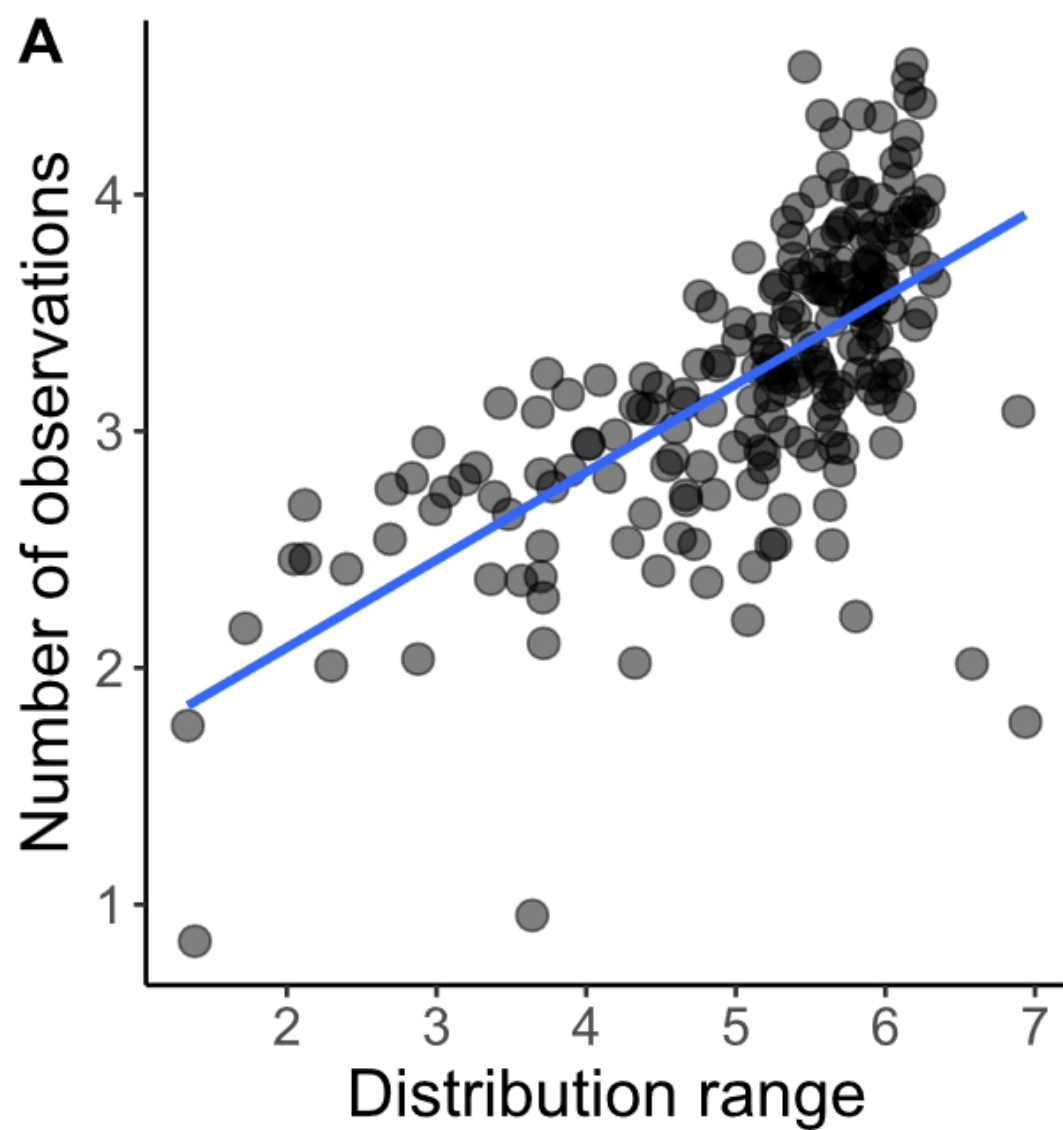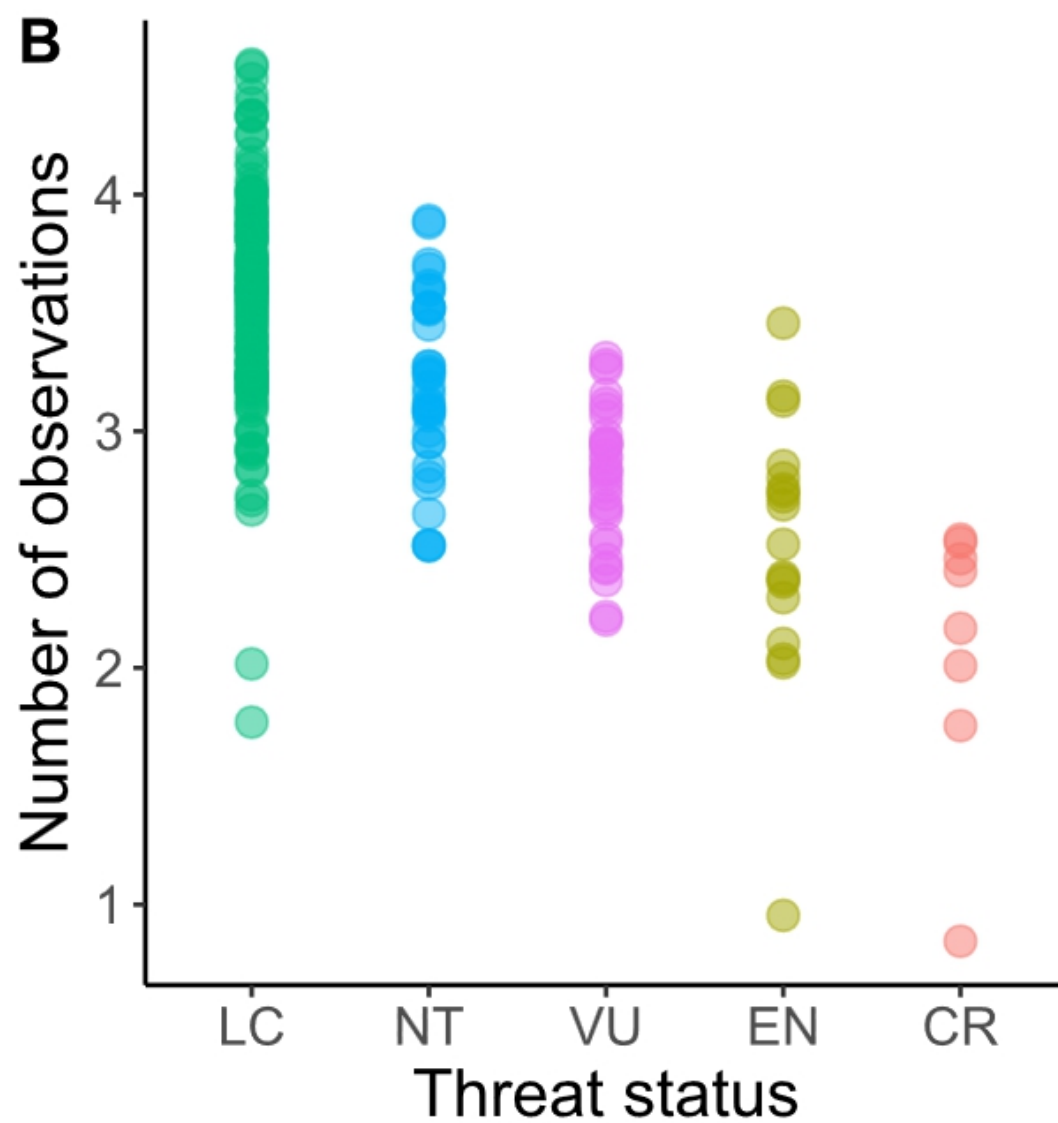

Supplement: Forti et al. supplementary material [file S2755095824000226sup001.zip › fig 1.pdf]
